# Natural Clay‐Based Materials for Energy Storage and Conversion Applications

**DOI:** 10.1002/advs.202004036

**Published:** 2021-03-24

**Authors:** Ye Lan, Yiyang Liu, Jianwei Li, Dajun Chen, Guanjie He, Ivan P. Parkin

**Affiliations:** ^1^ Department of Chemistry University College London 20 Gordon Street, WC1H 0AJ London UK; ^2^ State Key Laboratory for Modification of Chemical Fibers and Polymer Materials College of Materials Science and Engineering Donghua University Shanghai 201620 P. R. China; ^3^ School of Chemistry University of Lincoln Brayford Pool Lincoln LN6 7TS UK

**Keywords:** batteries, clays, energy storage and conversion, fuel cells, solar cells, supercapacitors

## Abstract

Among various energy storage and conversion materials, functionalized natural clays display significant potentials as electrodes, electrolytes, separators, and nanofillers in energy storage and conversion devices. Natural clays have porous structures, tunable specific surface areas, remarkable thermal and mechanical stabilities, abundant reserves, and cost‐effectiveness. In addition, natural clays deliver the advantages of high ionic conductivity and hydrophilicity, which are beneficial properties for solid‐state electrolytes. This review article provides an overview toward the recent advancements in natural clay‐based energy materials. First, it comprehensively summarizes the structure, classification, and chemical modification methods of natural clays to make them suitable in energy storage and conversion devices. Then, the particular attention is focused on the application of clays in the fields of lithium‐ion batteries, lithium–sulfur batteries, zinc‐ion batteries, chloride‐ion batteries, supercapacitors, solar cells, and fuel cells. Finally, the possible future research directions are provided for natural clays as energy materials. This review aims at facilitating the rapid developments of natural clay‐based energy materials through a fruitful discussion from inorganic and materials chemistry aspects, and also promotes the broad sphere of clay‐based materials for other utilization, such as effluent treatment, heavy metal removal, and environmental remediation.

## Graphical abstract

Natural clays have a broad range of application in energy and environmental fields. This work reviews the recent work of natural clays in the structure, classification, functionalization, and application in energy storage and conversion fields, and offers potential development strategies for clay‐based materials. The modified clays exhibit significant potentials as electrode materials, electrolytes, membranes, and nanofillers for energy devices.

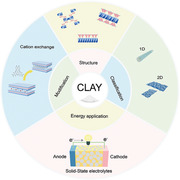

## 1 Introduction

With the increasing growth of the global economy, energy consumption, and environmental pollution are two major problems that face current society. Therefore, it is necessary to develop clean and sustainable energies, such as solar, wind and tidal energies. However, these renewable energies have intermittent and seasonal issues, thus making them difficult to satisfy demands of the daily life. Therefore, constant and efficient energy storage and conversion systems are required to be developed. The secondary batteries and supercapacitors, as major energy storage technologies, have high energy density and power density, respectively. The electrode materials, electrolytes and separators are vital components for energy storage systems. In addition, fuels cells and solar panels are powerful energy conversion techniques, they can be integrated with the energy storage devices to expand the utilization of the renewables. Due to the wide use and high demands of energy application, it is crucial to develop cheap, abundant and effective materials. Clay‐based materials are typical candidates exhibiting all these properties and are promising materials to be used in the energy storage and conversion field.

**Figure 1:**
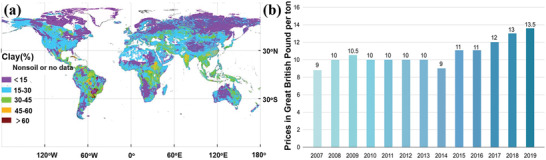
a) The global distribution of clay contents.^[^ ^1^ ^]^ b) The price trend of clay over the past 13 years. Reproduced with permission.^[^ ^1^ ^]^ Copyright 2014, John Wiley and Sons.

Natural clays are abundant all over the world. Their distribution is shown in **Figure** **1**a. As noted, high contents of clays emerge in the area of South America, Central Africa, India, and East Australia.^[^ ^1^ ^]^ Clay minerals form over long periods as a result of the gradual chemical weathering of rocks on the earth's surface. The weathering procedures include physical degradation and chemical disintegration, which convert initial minerals into clays. The influential factors controlling rock weathering process involve original types of rocks, water contents of rocks, temperature and weathering periods (https://pubs.usgs.gov/info/clays/). Different types of clays generally have different colors from white, light grey to red‐brown and orange‐red. The average prices of clay minerals are relatively low but have shown a gradually increasing trend over the last 13 years, increasing from £9 to £13.5 per tons, as shown in Figure 1b (https://www.statista.com/statistics/248190/average‐price‐of‐common‐clay/). The inorganic natural clay is typically composed of aluminum and magnesium silicate. At a chemical level, the silicate laminates consist of repeats of a silicon‐oxygen tetrahedron and an aluminum–oxygen octahedron.^[^ ^2^ ^]^ The silicate laminates are categorized into 1:1 type and 2:1 type. As for the dioctahedral clay, trivalent metals are dominant in the metal‐oxygen octahedron. But regarding to the trioctahedral clay, most of the divalent or monovalent metals occupy the center of the octahedron, such as saponite, hectorite and serpentine. Due to the robust mechanical properties and high chemical and thermal stability,^[^ ^3^ ^]^ natural clays have been widely used in the fields of liquid fertilizers, thickeners, stabilizers, drilling mud, fillers and food packaging.^[^ ^4^, ^5^, ^6^ ^]^ Additionally, natural clays have moderate to high specific surface area (1–750 m^2^ g^−1^)^[^ ^7^, ^8^ ^]^ and porous structure (porosity between 51% and 58%) (https://en.wikipedia.org/wiki/Porosity#Porosity_of_rocks), which can be reasonably applied as crucial materials in the fields of energy and environmental fields, such as absorbents and energy storage and conversion systems.

A majority of previous reviews of natural clays were mainly around environmental application such as dye effluent treatment, heavy metal removal and environmental remediation.^[^ ^7^, ^9^, ^10^ ^]^ Recently, multiple research works have been conducted about modified clays in the fields of energy storage systems, primarily with the focus on batteries (anodes, cathodes, separators, electrolytes),^[^ ^11^, ^12^, ^13^, ^14^ ^]^ supercapacitors,^[^ ^15^ ^]^ solar cells,^[^ ^16^ ^]^ and fuel cells,^[^ ^17^ ^]^ respectively. Ummartyotin's group has generally reviewed clay‐based materials in energy storage and conversion application with the focus on the dielectric and dye sensitized solar cell applications, which will not be summarized in detail herein.^[^ ^2^ ^]^

Due to the unique physicochemical characteristics of clays, they have attracted much research interest in fields of energy storage and conversion applications. Furthermore their low cost and natural abundance, together with their stable thermal, mechanical, and fire resistance properties make them excellent choices for large‐scale devices. Owing to their porous structures and high surface area, clays have large contact surfaces with the electrolyte, provide adequate active sites and typically enable fast ion diffusion. In addition, clays typically possess high ionic conductivity and excellent hydrophilicity, which are beneficial properties when used as solid‐state electrolytes or separators for aqueous battery application.

However, raw clay minerals are brittle and the structures of clays are inclined to aggregate during the charge/discharge process. The electronic conductivities of clays are low, inhibiting the fast transfer of electrons. Specially, clay derivative‐based anode materials for lithium‐ion batteries (LIBs) have a severe volume change during the charge/discharge process, resulting in the pulverization and structural fracture of the clay. In view of the above shortcomings of clays, appropriately decorated materials and modification methods should be used to improve the properties of clays in the energy field.

The development of clays as practical energy storage and conversion materials is rapid but not mature. Clay‐based materials have tremendous potential to become a type of burgeoning energy storage and conversion materials after the optimization of electrochemical properties. Hence, it is essential to summarize updated research progress of clay‐based energy materials. In this review, many up‐to‐date works and insights of clay‐based energy materials are comprehensively provided. First, the structure, classification, chemical and physical modification methods and physicochemical properties of clays are discussed. Secondly, a range of electrochemical applications of porous clays are introduced, such as LIBs, lithium–sulfur (Li–S) batteries, zinc‐ion batteries (ZIBs), chloride‐ion batteries (CIBs), supercapacitors, solar cells and fuel cells. Furthermore, different treatment methods are outlined to improve the electrochemical properties of clay‐based energy materials. Finally, we conclude the approaches for the modification of this emerging energy materials and their possible future development strategies. The aim of this review is to provide a directive perspective of clay‐based energy materials.

## 2 Structure and Classification of Clays

**Figure 2:**
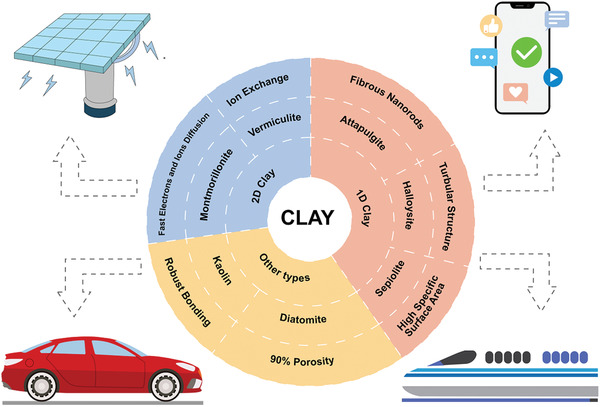
Classification, structure, and applications of the natural clay.

In this section, we will review the main structure and classification of clays with different dimensions. Natural clay minerals possess almost similar elemental compositions and crystalline structures. The silica and alumina contents occupy a major component of the clay. The oxides of magnesium, iron, calcium, sodium and potassium occupy minor proportion of different types of clays.^[^ ^18^ ^]^ According to structural differences, natural clay is generally divided into three types: 1D clay, 2D clay and other types, and they can be applied in different energy storage devices, which are summarized in **Figure** **2**. The basic information of the natural clays with different dimensions is displayed in **Table** **1**.

**Table 1:** The basic information of natural clays with different dimensions

Dimension	Clay type	Main elemental composition [% weight]	Density [g cm^−3^]	Formula weight [g mol^−1^]	Surface area [m^2^ g^−1^]	Schematic illustration^a)^	SEM morphology	Ref.
1D	ATP	SiO_2_ (58.38) Al_2_O_3_ (9.50)	2.1–2.3	583.38	130	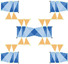	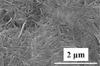	^[^ ^150^ ^]^
1D	Sepiolite	SiO_2_ (55.21) Al_2_O_3_ (0.43) Fe_2_O_3_ (0.15)	2.0–2.5	300.92	122	‐	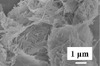	^[^ ^20^ ^]^
1D	Halloysite	SiO_2_ (46.86) Al_2_O_3_ (34.10) Fe_2_O_3_ (2.27)	2.0–2.2	252.24	20	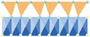	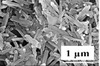	^[^ ^21^, ^151^ ^]^
2D	MMT	SiO_2_ (65.34) Al_2_O_3_ (12.89) Fe_2_O_3_ (2.38)	2.0–2.7	282.21	249	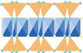	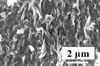	^[^ ^152^ ^]^
2D	Vermiculite	SiO_2_ (39.00) Al_2_O_3_ (12.00) Fe_2_O_3_ (8.00)	2.4–2.7	‐	10	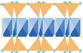	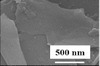	^[^ ^153^ ^]^
Other types	Kaolin	SiO_2_ (53.70) Al_2_O_3_ (43.60) Fe_2_O_3_ (2.00)	2.5–2.6	258.00	359	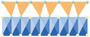	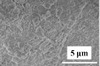	^[^ ^154^ ^]^
Other types	Diatomite	SiO_2_ (72.00) Al_2_O_3_ (11.40) Fe_2_O_3_ (5.80)	0.5	60.00	1	–	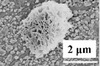	^[^ ^28^ ^]^

^a)^ The blue part represents an octahedral aluminum magnesium hydroxide nanosheet. The yellow part represents a tetrahedral silica nanosheet.

### 2.1 1D Clay

In this section, we aim to give an overview of the structures and morphologies of the 1D clays. 1D natural clays mainly include attapulgite (ATP), sepiolite and halloysite. The ATP clay possesses a porous architecture and its fibrous nanorods are disorderly stacked. The monocrystalline structure of ATP is 20–50 nm in diameter and 0.5–1 µm in length.^[^ ^11^, ^19^ ^]^ ATP has many attractive physical properties, such as large specific surface area (130 m^2^ g^−1^), high adsorption capacity, stable chemical and thermal properties. These important physical properties make the ATP clay to absorb more electrolytes, and increase the safety of the energy devices. While, the sepiolite clay possesses a consecutive tetrahedral nanosheet and lacks a consecutive octahedral nanosheet. The sepiolite clay with fibrous structure displays an average pore diameter of 0.54 nm.^[^ ^20^ ^]^ The high specific surface area (122 m^2^ g^−1^) of sepiolite clay provides more active structures. Sepiolite is prone to conglomerate due to hydrogen bonding interaction between the sepiolite nanorods. Halloysite clay exhibits natural tubular morphology with the diameter of 5–20 nm and the tube length of 0.5–10 µm.^[^ ^21^ ^]^ The interlayer of halloysite is Al‐OH nanosheets and covered with Si—O nanosheets, while the external surface of which is primarily Si—O—Si and Si—OH groups. Halloysite clays have double‐layered structure with the open ends and inner lumen.^[^ ^22^ ^]^ Halloysite shows lower surface area (20 m^2^ g^−1^) compared to other 1D clays. Therefore, the interconnected pores stemming from random and accumulated 1D nanorods may alleviate the volume expansion of the clay during charge storage processes. The porous nanorods of the 1D clay could absorb more electrolytes into the structure and promote ion and electron diffusion, thus contributing to excellent cycling and rate performance.

### 2.2 2D Clay

In this section, we will review the structures and morphologies of the 2D clays. 2D natural clays mainly include montmorillonite (MMT) and vermiculite. The chemical formula of MMT is (Na,Ca)_0.33_(Al,Mg)_2_(Si_4_O_10_)(OH)_2_·nH_2_O. The 2:1 layers of MMT consist of alumina octahedral nanosheets located between two tetrahedral nanosheets.^[^ ^3^, ^7^ ^]^ The MMT clay has high specific surface area (249 m^2^ g^−1^), and the nanosheets tend to aggregate into a flat surface. The bonding within the MMT layers is relatively weak and allow ions to freely transport within the interlayer, which causes favorable rate properties. Vermiculite clays have a 2:1 type construction. The porous vermiculite clay has layered structure and high pore volume.^[^ ^23^ ^]^ The shrinkage and expansion of vermiculite's interlayers enable a high cation‐exchange capacity. It is generally known that 2D energy storage materials could help to shorten the ion diffusion pathway.^[^ ^24^ ^]^ Furthermore, 2D clays could provide sufficient intercalated sites and outstanding charge storage ability, which result in large contact area between the electrodes and electrolytes and short ion transportation pathways.^[^ ^25^ ^]^

### 2.3 Clay of Other Types

Kaolin clays show different morphologies with nanosheets or nanotubes in different regions.^[^ ^26^ ^]^ The kaolin clay consists of one silica tetrahedral nanosheet connected to an alumina octahedral nanosheet. Thus, the kaolin clay is categorized as 1:1 type clay. The interlayer bonding consisting of van der Waals forces and hydrogen bonds is surprisingly robust, therefore the interlayer of kaolin clays generally cannot be expanded. The kaolin clay has the highest specific surface area (359 m^2^ g^−1^) among above listed clays in Table 1. The kaolin clay has excellent chemical and thermal stability, as well as an extensive distribution around the world.^[^ ^27^ ^]^ Diatomite clays have two different types, including disk type and linear type.^[^ ^28^ ^]^ Diatomite clays possess low density and huge adsorption capacity. Diatomite clays contain 72% of silica, and the porosity of which is approximately 90%. Thus, it has good adsorption ability for liquid electrolytes.

The important physical properties of clays as separators and electrolytes are their ionic conductivity and porosity. The physical properties of clay‐based composites are summarized in **Table** **2**. The high ionic conductivity could boost the transportation of lithium ions and decrease the interfacial resistance. Furthermore, the superior porosity could increase the electrolyte uptake and maintain sufficient charge carriers between the electrodes, further improving the cycling stability. Nevertheless, the ionic conductivity and porosity of pure clays are not provided in previous research, which impede the subsequent development of clay‐based energy materials. Therefore, the study of clay‐based energy materials needs to be more targeted and selective.

**Table 2:** The conductivity and porosity of clay‐based composites as energy materials

Clay type	Application	Composite clay‐based energy materials	Ionic conductivity	Porosity	Ref.
MMT	Separator	PVDF/poly(vinylpyrrolidone)/nanoclay	5.61 mS cm^−1^	87%	^[^ ^155^ ^]^
Halloysite	Separator	Bacterial cellulose/halloysite nanotubes	5.13 mS cm^−1^	83%	^[^ ^88^ ^]^
Dickite	Separator	Expanded dickite on cross‐linked nonwoven fabrics	3.16 mS cm^−1^	62%	^[^ ^31^ ^]^
Halloysite	Solid polymer electrolyte	Clay composited methoxy poly(ethylene glycol) acrylate polymer	5.62 × 10^−5^ S cm^−1^	‐	^[^ ^14^ ^]^
MMT	Solid‐state electrolyte	MMT/dimethyl sulfoxide nanocomposites	2.00 × 10^−4^ S cm^−1^	‐	^[^ ^106^ ^]^
Halloysite	Electrolyte	PEO+ LiTFSI + halloysite nanotube	1.11 × 10^−4^ S cm^−1^	‐	^[^ ^101^ ^]^
Halloysite	Gel polymer electrolytes	Cellulose acetate/poly‐L‐lactic acid/Halloysite nanotube composite membranes	1.52 × 10^−3^ S cm^−1^	83%	^[^ ^104^ ^]^

As discussed above, the clays with different dimensions have the similar chemical compositions. The clays with 1D, 2D, and other types of structures possess considerable specific surface area and porous structure, which lead to superior performances in energy storage and conversion devices. More importantly, compared with clays with 1D nanorod and nanotube structure, clays with 2D structure contribute to good stabilization between interfaces and nanostructures, thus reducing volume change during charge/discharge processes. 2D clays may not break into small particles and maintain intact during cycling, indicating better structural stability than 1D clays.

## 3 Modification Methods of Clays

As stated in the previous section, natural clays have different structures with the focus of 1:1 type and 2:1 type. Furthermore, clays with different dimensions were classified into three types. Chemical modification methods were utilized to avoid the drawbacks of the initial structures of clays for a specific use. In this section, we will further review the different modification methods of clays. The modification methods of clays are diverse, which primarily depend on the required structure and prospective properties. To improve the electrochemical properties of clays in different energy storage and conversion fields, targeted modification methods are summarized to optimize the structure of clays.

### 3.1 Acid Leaching

Basically, natural clays contain many impurities, such as metal oxides, quartz, and silts, and thus acid leaching is an essential process. Zhu et al. utilized 1 mol L^−1^ HCl solution to wash crude ATP, in order to remove the impurities in clays.^[^ ^29^ ^]^ Furthermore, Wang et al. explained the effect of humic acid modification on the interlayer properties of MMT.^[^ ^30^ ^]^ After adding humic acid, the interlayer spacing of the clay decreased, thus reducing the water content within the interlayer. Humic acid was selected to enhance the immobilization performance and the long‐term effectiveness of the clay‐based soil stabilization process. However, the acid leaching could remove general metal oxide impurities, excluding quartz. After acid leaching process, metal ion impurities should be washed by deionized water and purified clay materials were obtained.

### 3.2 Expansion of Interlayer Spacing

The interlayer spacing of raw clay should be further enlarged for fast ion diffusion during the energy storage processes. Thus, the concept of expanded clay with an exfoliated structure was proposed. The loose structure of expanded clay has the ability to offer fluent ion diffusion channels and high porosity, thus improving electrolyte uptake. For example, Liu et al. calcined the dickite‐urea composite and potassium chlorate, and the obtained expanded dickite clay showed an adjustable pore structure (porosity from 32.9% to 61.6%).^[^ ^31^ ^]^ The influence of the mass ratio of urea and potassium chlorate on the pore morphology of the dickite was revealed. When the mass ratio of external urea to potassium chlorate was 0.448, the interlayer spacing of the expanded dickite was 0.212 µm. This method developed a tunable morphology of clays, but the preparation process was complicated and time‐consuming. The external urea needed to be removed and the excessive ethanol needed to be evaporated.

### 3.3 Cationic Exchange

The mechanism of the cationic exchange reaction is related to the electrostatic attraction between negatively charged surface of the clays and cationic surfactants. The cationic head groups of the polymer were preferentially absorbed on the surface of the clay layers, while the cationic segments extend the clay interspace distance and transform the initial hydrophilic clays into organoclays.^[^ ^32^ ^]^ The dispersion of clays into polymer matrix displayed noteworthy improvement on mechanical properties, such as shear modulus, as well as thermal properties, such as glass transition temperature.^[^ ^2^ ^]^ The above results indicated that the cationic exchange endowed the natural clays with more favorable mechanical and thermal properties and the enlarged interspace for absorbing more electrolytes. Concerning the good cationic exchange ability of crystal violets, Chen et al. intercalated MMT with crystal violet.^[^ ^33^ ^]^ This organoclay provided the sources of silicon, carbon, and nitrogen for lithium‐ion battery anodes. Notably, the organoclay displayed a hierarchical porous structure and a high specific surface area of 153 m^2^ g^−1^. Therefore, after the cationic exchange reaction, clay composites obtained desirable properties by adjusting types of cationic polymers used. Whereas, there are increasing concerns about compatibility between most of the hydrophobic polymer monomers and the hydrophilic clay.

### 3.4 Wettability Modification

Clays modified by hydrophilic polymers, such as polyacrylic acid and polyvinyl alcohol (PVA), will improve their hydrophilicity. For example, Zhu et al. fabricated PVA modified ATP‐based nanofibrous membranes.^[^ ^34^ ^]^ The effects of PVA contents on the pore structure, hydrophilic properties and mechanical strength of ATP‐based nanofibrous membranes were studied. The outstanding hydrophilic property was due to the porous morphology of the clay and hydroxyl groups of PVA when exposed on the surface of the membrane. After a sinter process of materials with the extra PVA, the clay‐based nanofibrous membranes had a high porosity (above 60%), excellent flexural strength (5–7 MPa), improved hydrophilicity (water contact angle of 20.3°) and remarkable chemical stability for effective oil/water separation. As for aqueous batteries, hydroxyl groups derived from modified clays could have a strong interaction with water molecules from the electrolyte. Therefore, improved hydrophilic clays maintain the high electrolyte uptake and stable cycling performance. Hence, it is necessary to modify the wettability of clays when used in aqueous batteries. Although the wettability modification method improves the hydrophilicity of clays, its applications in different energy storage devices should be further adjusted and the correlations between the modification of wettability and electrochemical performances should be considered systematically.

### 3.5 Other Methods

The calcination treatment of clays is commonly adopted to eliminate the organic phases or any impurities. Dong et al. calcinated Congo red‐loaded 2D layered double hydroxide clays, and fabricated new compounds with optimized architecture and more active adsorption sites for contaminants.^[^ ^35^ ^]^ This was facilitated due to the decomposition and dehydration of hydroxyl and carbonate groups of the composite clays during the calcination process. Additionally, the raw clay modified with pore‐forming agent significantly enhance the specific surface area of the clay.^[^ ^36^ ^]^ Daud et al. prepared porous clays with a pore‐forming agent – starch powder.^[^ ^37^ ^]^ When the content of starch powder was 30 vol%, the porosity of clays was 30%. The high porosity allowed high proton transfer for separators in microbial fuel cells, thus improving power output.

Therefore, the morphology and structure of clays will be regulated through different modification methods. The versatile modification methods of the clays result in the structural optimization and the desirable performance.

## 4 Applications of Clays

In the Sections 2 and 3, we have reviewed the main structures, classifications, and modification methods of clays. The modification methods of clays render them structural optimization. The special structure and physicochemical properties of clays result in promising application prospects. In this section, we will review some major applications of modified clays in the fields of energy storage and conversion, which we have generally categorized into three domains: clay‐based composites in rechargeable metal‐ion batteries (Section 4.1), clay‐based composites for supercapacitors (Section 4.2), and clay‐based composites for energy conversion systems (Section 4.3).

### 4.1 Clay‐Based Composites in Rechargeable Batteries

#### 4.1.1 Anodes

One of the dominating applications of clay‐based energy material is electrode materials for rechargeable metal‐ion batteries. The secondary batteries include monovalent ion batteries (lithium, sodium and potassium), divalent ion batteries (zinc, magnesium, calcium) and trivalent ion batteries (aluminum). The batteries consist of cathodes, anodes, separators and electrolytes. The anode materials used in rechargeable metal‐ion batteries are commonly metals and graphite.^[^ ^38^ ^]^ The lithium metal has a high theoretical specific capacity (3860 mAh g^−1^, 2061 mAh cm^−3^) and the most negative reduction potential (−3.04 V vs the standard hydrogen electrode).^[^ ^39^, ^40^ ^]^ However, dendrite formation and huge volumetric change of the lithium metal can occur during charge/discharge processes,^[^ ^41^ ^]^ which seriously influence overall electrochemical properties and the safety.

Clays typically have good rigidity, affinity to lithium, and high specific surface area to accommodate lithium, and thus clays are appropriate candidates to become the host materials for lithium. Zhou et al. reported that diatomite could serve as an ideal host for constructing silicon‐lithium based hierarchical anodes.^[^ ^42^ ^]^ The composite anode exhibited excellent cycling stability (0.04% capacity decay after 500 cycles at 0.5C) and favorable rate performance (65 mAh g^−1^ at 5C). Zhang et al. fabricated Na^+^‐pillared NiFe‐saponite (NF‐SAP) anode materials for LIBs.^[^ ^43^ ^]^ Saponites had a large interlayer spacing and favorable ion intercalation capacity for electrochemical processes. The pre‐pillaring of Na^+^ endowed NF‐SAP a larger interlamellar spacing of 11.6 Å to increase the active surface area. The metallic elements Fe and Ni underwent valence state changes during the cycling process. The NF‐SAP/Li half‐cell exhibited a specific capacity of 815 mAh g^−1^ during the 350th cycle at the current density of 500 mA g^−1^. Therefore, natural clays could act as potential templates for synthesizing hierarchical hybrid anodes for high‐performance batteries.

##### Clay Derivatives for LIB Anodes (Silicon)

Compared with metal lithium anode, silicon‐based anodes have a higher theoretical capacity of 4200 mAh g^−1^ and a low lithiation potential (0.2 V vs Li/Li^+^).^[^ ^44^ ^]^ The clay, as a typical abundant silicon source, has been applied as an anode material for LIBs. The reaction mechanism of Si anodes for LIBs was unraveled by Kitada's group:^[^ ^45^ ^]^ the Si anode is crystalline‐silicon (c‐Si) before the lithiation reaction. During the initial lithiation process, c‐Si transforms to amorphous‐Li_x_Si (a‐Li_x_Si). Then, in a completely lithiated state, c‐Li_15_Si_4_ is formed as shown in the Equations (1) and (2). The c‐Li_15_Si_4_ is converted to a‐Li_x_Si in the process of delithiation and the a‐Si after a completed delithiation, shown in the Equations (3) and (4)

Initial lithiation:

(1) $$c-\mathrm{Si}+xLi^{+}+xe^{-}\to a-Li_{x}\mathrm{Si}$$

(2) $$4a-Li_{x}\mathrm{Si}+yLi^{+}+ye^{-}\to \;c-Li_{15}Si_{4}$$

Subsequent lithiaion/de‐lithiation:

(3) $$c-Li_{15}Si_{4}+zLi^{+}+ze^{-}↔4a-Li_{x}\mathrm{Si}$$

(4) $$a-\mathrm{Si}+xLi^{+}+xe^{-}↔a-Li_{x}\mathrm{Si}$$

Although silicon‐based anodes possess a high capacity, they suffer from severe volumetric expansion (≈ 300%) and inferior electronic conductivity (6.7 × 10^−4^ S cm^−1^).^[^ ^46^, ^47^, ^48^ ^]^ The huge volumetric change results in the formation of unstable solid electrolyte interphase (SEI) and exfoliated Si from current collectors, ultimately leading to a fast decay of capacity and a limited cycling lifespan.^[^ ^49^ ^]^ The poor electronic conductivity and low ion diffusion efficiency of the Si anode result in an unsatisfactory rate performance.^[^ ^50^ ^]^

Reducing the size of silicon to the nanoscale is an effective way to solve the problem. Zhou et al. extracted interconnected Si nanoparticles from natural halloysite through acid etching and magnesiothermic reduction.^[^ ^51^ ^]^ The sulfuric acid solution was used to remove the alumina sheets from halloysite. Then, the magnesiothermic reduction reaction was carried out at 650–700 °C, shown in the Equation (5)

(5) $$\mathrm{Si}O_{2}+2\mathrm{Mg}\to \mathrm{Si}+2\mathrm{MgO}$$

The diameter of the obtained Si nanoparticles was 20–50 nm. Due to the nano size and porous structures, Si anodes offered open channels for the uptake of electrolytes and had the space to accommodate the volumetric change. Therefore, Si anodes exhibited a high specific capacity above 800 mAh g^−1^ at a current density of 1C after 1000 cycles.

**Figure 3:**
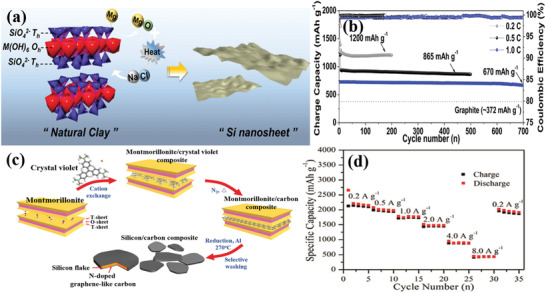
a) Schematic illustration for the preparation process of 2D Si nanosheet.^[^ ^52^ ^]^ b) Long‐term cycling stability of carbon coated Si nanosheets anode at different rates. Reproduced with permission.^[^ ^52^ ^]^ Copyright 2016, American Chemical Society. c) Schematic illustration for the preparation process of silicon flake/nitrogen‐doped graphene‐like carbon anode.^[^ ^33^ ^]^ d) Rate performance of silicon flake/nitrogen‐doped carbon anode. Reproduced with permission.^[^ ^33^ ^]^ Copyright 2019, Royal Society of Chemistry.

Another effective approach is using a carbon layer coating to enhance the electronic conductivity and structural integrity of clay‐based anodes. Ryu et al. fabricated 2D Si nanosheets from the MMT clays through a one‐step molten salt‐induced exfoliation and chemical redox process,^[^ ^52^ ^]^ as illustrated in **Figure** **3**a. The natural clays reacted with metal reductants in the molten salt matrix (Mg + NaCl), which simultaneously serve as an exfoliating agent and a heat scavenger. This one‐step preparation method produced 5 nm‐thickness porous Si nanosheets. The specific surface area of the Si nanosheets was 308 m^2^ g^−1^. Moreover, the pore size of the Si nanosheets was ≈20 nm. Acetylene gas served as the carbon source in the process. The addition of a carbon layer enhanced the electron conductivity and buffered the volumetric expansion of the Si nanosheets. The carbon coated Si nanosheet anode had an excellent capacity retention of 91.7% and high Coulombic efficiency after 700 cycles at 1.0C, as shown in Figure 3b.

Chen et al. reported an innovative synthetic process for the preparation of silicon flakes from the MMT via low‐temperature aluminothermic reduction reaction,^[^ ^33^ ^]^ as demonstrated in Figure 3c. The crystal violet served as the carbon source and was added into MMT clays via a cation exchange reaction. The silicon flake/nitrogen‐doped graphene‐like carbon composite displayed a hierarchical porous structure, a high specific surface area (153 m^2^ g^−1^) and a lamellar morphology. The 2D silicon flakes could withstand the diffusion‐induced stress. The nitrogen‐doped carbon coating further enhanced the electronic conductivity and provided more active sites toward lithium storage. Owing to the synergetic functions, the silicon flake/nitrogen‐doped carbon anode showed a remarkable cycling performance (reversible specific capacity of 1138 mA h g^−1^ at 1.0 A g^−1^ after 240 cycles). As shown in Figure 3d, the composite anode displayed an outstanding rate performance (specific capacity of 2187 mA h g^−1^ at 0.2 A g^−1^ and 434 mA h g^−1^ at 8.0 A g^−1^).

**Figure 4:**
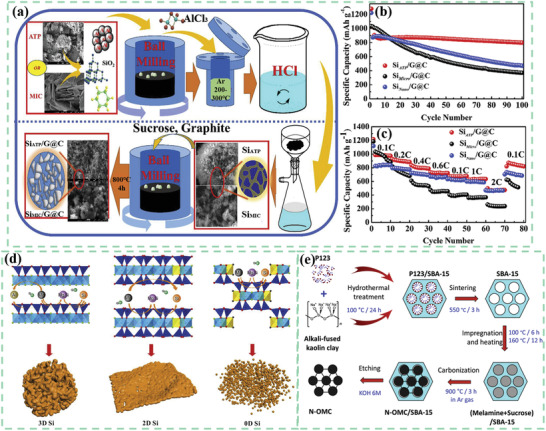
a) Schematic illustration for the preparation process of Si_ATP_/G@C and Si_MIC_/G@C.^[^ ^53^ ^]^ The electrochemical properties of Si_ATP_/G@C, Si_Micro_/G@C and Si_Nano_/G@C: b) cycling performance and c) rate capability. Reproduced with permission.^[^ ^53^ ^]^ Copyright 2019, Elsevier. d) Schematic illustration for the preparation process of different types of clays generated nano Si with different morphologies.^[^ ^54^ ^]^ Reproduced with permission.^[^ ^54^ ^]^ Copyright 2018, Elsevier. e) Schematic illustration for the preparation process of N‐doped ordered mesoporous carbon utilizing the kaolin clay as the template.^[^ ^55^ ^]^ Reproduced with permission.^[^ ^55^ ^]^ Copyright 2020, Elsevier.

Gao et al. prepared porous Si/graphite@carbon (Si/G@C) anode materials through a low‐temperature aluminothermic reduction reaction, shown in **Figure** **4**a.^[^ ^53^ ^]^ The silicon was derived from natural ATP. The specific surface area of porous Si_ATP_/G@C was 210.2 m^2^ g^−1^ and the pore size distribution was centered at 3 µm. As shown in Figure 4b,c, in contrast with micro‐Si/graphite@carbon (Si_Micro_/G@C) and nano‐Si/graphite@carbon (Si_Nano_/G@C), Si_ATP_/G@C showed outstanding electrochemical properties. The Si_ATP_/G@C displayed an excellent cycling stability with a high reversible capacity of 799 mAh g^−1^ at a current density of 100 mA g^−1^ after 100 cycles and great rate performance with a specific capacity retention of 470 mAh g^−1^ when the current density increased to 2C. The successful preparation of porous Si_MIC_ from mica (MIC) powders indicated that the novel fabrication method was suitable to other types of clays. As expected, the Si_MIC_/G@C anode also demonstrated an outstanding cycling performance with a specific capacity of 697 mAh g^−1^ after 100 cycles. The universal method of low‐temperature aluminothermic reduction combined with an inexpensive natural clay increases the practicality of clay‐based anodes.

Chen et al. concluded that the morphology of the prepared nanostructured Si relied on the types and architectural characteristics of the original clays.^[^ ^54^ ^]^ For example, 0D nano Si derived from ATP, 2D nano Si stemmed from MMT, and 3D nano Si derived from halloysite, shown in Figure 4d. From the XRD result the crystal sizes of Si derived from ATP, MMT, and halloysite were 90, 49, and 24 nm, respectively. The specific surface areas of Si (ATP), Si (MMT), and Si (halloysite)were around 116, 83, and 80 m^2^ g^−1^, respectively. The 2D nanostructure stemmed from MMT could reinforce the stabilization of the interface, thus buffering the huge volumetric variation and retaining the structural integrity during charge/discharge processes. Moreover, the interconnected characteristic of 2D Si resulted in fast electron and ion diffusion and restricted the irreversible depletion of lithium ions. The relationship between the structure and the electrochemical property of LIBs was also disclosed, whereby 2D nano Si from MMT displayed the best electrochemical performance (1369 mAh g^−1^ at 1.0 A g^−1^ over 200 cycles). Furthermore, natural clays were not only applied in active anode materials for LIBs, but also regarded as an ordered mesoporous hard template.

Le et al. fabricated N‐doped ordered mesoporous carbon by etching the silica template method utilizing kaolin clay (the fabrication process was shown in Figure 4e).^[^ ^55^ ^]^ The amorphous and ordered mesoporous silica SBA‐15 template was synthesised through solid state reaction with NaOH. The specific surface area of SBA‐15 was 635.2 m^2^ g^−1^ and the pore size varied from 4.1 to 13.9 nm. The large specific surface area of SBA‐15 promoted the remarkable capacity (648.7 mA h g^−1^ at a current density of 0.1 A g^−1^ after 100 cycles) and rate performance (672 mA h g^−1^ at 0.5 A g^−1^ and 358 mA h g^−1^ at 5 A g^−1^) of N‐doped ordered mesoporous carbon anodes. As discussed above, reducing the size of silicon and coating carbon layer are effective approaches to improve the electrochemical performances of silicon‐based anodes but current methods are complicated and involve multi‐steps, and thus simpler methods can be developed for these modifications in the future research.

##### Clay Derivatives for LIB Anodes (Silica)

In this section, we reviewed effective approaches to improve the electrochemical properties of clay‐derived silica‐based anode materials. Although Si materials derived from the clay have many merits for the anodes in LIBs, the extraction process can be complicated and time‐consuming. Compared with Si materials, the fabrication process of silica stemmed from clays neglects the reduction step. Significantly, the silica possesses relatively high theoretical specific capacity (1965 mAh g^−1^) and abundant reserves.^[^ ^56^ ^]^ The reaction between the silica and the lithium metal generates Li_2_O, Li_4_SiO_4_, and Si in the initial intercalation process.^[^ ^57^ ^]^ The first two inactive materials not only suppress the aggregation of Si, but also buffer the severe volumetric expansion of Si.

Lan's group employed a simple method to prepare the ATP/polyacrylonitrile precursor aerogel.^[^ ^11^ ^]^ Through the pre‐oxidized and carbonized treatment of the precursor aerogel, amorphous carbon coated silica anode materials were successfully prepared. The uniform porous structure of ATP could absorb more electrolytes into the active anodes and shorten lithium‐ion diffusion pathways. The continuous carbon structure enhanced the interface stability between electrode materials and electrolytes, which promoted the formation of a stable SEI layer. The synergistic function of the silica and the carbon layer contributed to a remarkable cycling stability and rate capability. The electrochemical reactions for inserting lithium ions are shown as follows (Equations ((6), (7), (8), (9)))^[^ ^56^ ^]^

(6) $$\mathrm{Si}O_{2}+4Li^{+}+4e^{-}\to \;2Li_{2}O+\mathrm{Si}$$

(7) $$2\mathrm{Si}O_{2}+4Li^{+}+4e^{-}\to \;Li_{4}\mathrm{Si}O_{4}+\mathrm{Si}$$

(8) $$5\mathrm{Si}O_{2}+4Li^{+}+4e^{-}↔\;2Li_{2}Si_{2}O_{5}+\mathrm{Si}$$

(9) $$\mathrm{Si}+xLi^{+}+xe^{-}↔\;Li_{x}\mathrm{Si}$$

The porous anodes exhibited an initial discharge specific capacity of 1628.4 mAh g^−1^ and an average discharge capacity of 534.6 mAh g^−1^ at 0.1 A g^−1^ after 50 cycles. As discussed above, the effective utilization of natural clays can reduce the cost and stimulate the sustainable development in the field of anode materials for LIBs.

##### Clays for Sodium Ion Battery (NIB) and Potassium Ion Battery (KIB) Anodes

Silicon‐based anodes derived from clay react with alkali ions (lithium, sodium and potassium) with an alloying mechanism.^[^ ^58^ ^]^ Compared with LIBs, NIBs and KIBs have attracted more attention in recent years, and clay‐based materials have promising application prospects. In this section, we reviewed the silicon and silica based anode materials for NIBs and KIBs. This is because the Earth reserves of the chemical elements Na and K are more than 1000 times than that of the metal Li.^[^ ^59^, ^60^ ^]^ Nevertheless, the theoretical capacities of Na (1166 mAh g^−1^) and K (685 mAh g^−1^) are far less than that of Li (3861 mAh g^−1^). Furthermore, the ionic radii of Na^+^ (1.02 Å) and K^+^ (1.38 Å) exceed that of the Li^+^ (0.76 Å), resulting in sluggish diffusion kinetics of Na^+^ and K^+^ in NIBs and KIBs. The silicon anode of LIBs exhibits a high specific capacity of 4200 mAh g^−1^ for Li_22_Si_5_ with complete lithiation and 3579 mAh g^−1^ for Li_15_Si_4_.^[^ ^61^, ^62^ ^]^ In addition, the alloying reactions between Na^+^, K^+^ and silicon display capacities of 954 mAh g^−1^ for NaSi and 995 mAh g^−1^ for KSi, respectively. The high capacity of silicon‐based anodes in LIBs are unparalleled with those in NIBs and KIBs. The volumetric changes of Si in NIBs and KIBs are 114% and 334%, respectively.^[^ ^58^ ^]^ Undesirably, the reversible insertion and extraction of Na^+^ and K^+^ are hindered by the poor diffusion kinetics. There are limited redox reactions between Si and Na^+^ or K^+^. As a consequence, the c‐Si anode is not relatively active toward Na^+^ and K^+^, but the a‐Si seems to be a potential candidate.^[^ ^58^, ^63^ ^]^ The a‐Si derived from clays could avoid structural fracture during cycling. Besides, the disordered atom arrangement in a‐Si is related to vacancies, which offer a fast diffusion pathway for electrons and ions.

##### Clays for Aqueous ZIB Anodes

In the above sections, the application of clay derivatives used in alkali metal ion batteries were reviewed. Alkali metal ion batteries with organic electrolytes are sensitive to humidity and oxygen, requiring rigorous fabrication processes and often leading to unsatisfactory safety issues; particularly because organic electrolytes are explosive and poisonous. In the meantime, aqueous electrolytes are more cost‐effective and show better ionic conductivity. As a potential alternative to LIBs, aqueous ZIBs have the advantages of low cost, high safety, high volumetric capacity (5855 mA h cm^−3^) and suitable redox potential (−0.76 V vs standard hydrogen electrode).^[^ ^64^, ^65^ ^]^ The ionic conductivity of aqueous electrolytes can reach to 1 S cm^−1^, which is better than that of flammable electrolytes in LIBs.^[^ ^66^ ^]^ However, the metal Zn anode suffers from sharp dendrite formation, side reactions, low Coulombic efficiency and a short cycling life.^[^ ^67^ ^]^ Worst of all, Zn dendrite can puncture the separator and cause a short circuit.^[^ ^68^ ^]^ Therefore, many approaches have been adopted to improve the surface structure of Zn anodes. The porous clay conducts ions and insulates electrons, which is in favor of impeding dendrite growth during Zn^2+^ migration.

**Figure 5:**
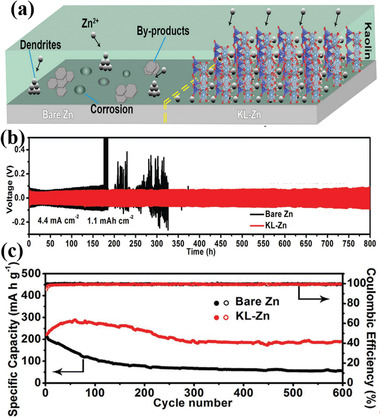
Bare Zn versus KL‐Zn. a) Schematic illustration of the morphology during Zn^2+^ deposition process.^[^ ^69^ ^]^ b) Long‐term galvanostatic cycling of the symmetrical cells at the current density of 4.4 mA cm^−2^. c) Long cycling performance of the MnO_2_ cells cycled with Zn and KL‐Zn anodes at the current density of 0.5 A g^−1^. Reproduced with permission.^[^ ^69^ ^]^ Copyright 2020, Wiley‐VCH.

Deng et al. exploited the kaolin clay coated onto the surface of Zn anodes (KL‐Zn).^[^ ^69^ ^]^ The kaolin clay is cheap (generally £30–200 per ton) and abundant worldwide. In contrast with the large charge‐transfer resistance of 735.7 Ω for pure Zn, KL‐Zn anodes with 21 µm coating layer demonstrated a lower resistance (70.35 Ω). Due to the uniform pore diameter distribution (≈3.0 nm) of kaolin, Zn^2+^ transportation was restricted by kaolin channels, thus achieving the dendrite‐free deposition. Compared with pure Zn anodes in **Figure** **5**a, the protective kaolin clay layer could effectively restrain the formation of zinc dendrites and other by‐products (such as Zn hydroxides and zincates) during Zn stripping/plating. The kaolin clays with sufficient active adsorption sites enhanced the affinity of Zn^2+^ and reduced the resistance of Zn nucleation. Due to the existence of sieve‐functional and porous kaolin clays, symmetrical cells had good cycling stability (800 h lifespan with low voltage hysteresis) and KL‐Zn/MnO_2_ batteries exhibited a high specific capacity (190 mA h g^−1^ after 600 cycles at 0.5 A g^−1^), shown in Figure 5b,c.

In a word, clay‐based anodes could serve as the silicon or silica source applied in the LIBs. From **Table** **3**, we could conclude that silicon anodes have higher specific capacity than silica anodes, which is consistent with theoretical specific capacities of silicon and silica. Nevertheless, the extraction process of silicon from clays is more complicated than that of silica. Moreover, clays could impede the growth of zinc dendrite in the field of ZIBs. However, c‐Si anodes derived from clay are not suitable in the fields of NIBs and KIBs according to current studies. Using a‐Si anode prepared from clay precursors is an effective method to address above issues. The utilization of clay‐based materials in aqueous battery anodes is still at the early stage, clay or clay derivatives anodes need to be explored in other aqueous battery systems.

**Table 3:** The electrochemical performances of different clay‐based anodes

Anode type	Specific capacity [mAh g^−1^]	Current density	Cycle numbers	Ref.
Si nanoparticles from natural halloysite (LIBs)	800	1.0 C	1000	^[^ ^51^ ^]^
Carbon‐coated Si nanosheet from MMT clays (LIBs)	865	1.0 A g^−1^	500	^[^ ^52^ ^]^
Silicon flake/nitrogen‐doped carbon from MMT (LIBs)	1138	1.0 A g^−1^	240	^[^ ^33^ ^]^
Si/graphite@carbon from ATP (LIBs)	799	0.1 A g^−1^	100	^[^ ^53^ ^]^
2D nano Si from MMT (LIBs)	1369	1.0 A g^−1^	200	^[^ ^54^ ^]^
Amorphous carbon coated silica from ATP (LIBs)	534.6	0.1 A g^−1^	50	^[^ ^11^ ^]^
Kaolin clay coated onto the surface of Zn anodes (ZIBs)	190	0.5 A g^−1^	600	^[^ ^69^ ^]^

#### 4.1.2 Cathodes

The modified clays can not only serve as the silicon or silica source of anodes (Section 4.1.1), but also the cathodes of Li–S batteries, ZIBs and CIBs. Li–S batteries have drawn much attention as alternatives to LIBs. As one of the most promising next‐generation batteries, Li–S batteries have a high energy density of 2567 Wh kg^−1^ and there is a high earth abundance of sulfur with a high theoretical capacity of 1675 mAh g^−1^.^[^ ^70^ ^]^ However, the sulfur cathode has a poor electron conductivity (5.0 × 10^−30^ S·cm^−1^), severe volumetric change (≈80%) and a shuttle effect of Li polysulfides during the charge/discharge process.^[^ ^71^, ^72^ ^]^ Many modified carbon materials were introduced to improve the electron conductivity and suppress the shuttle effects of polysulfides.^[^ ^73^ ^]^ However, the hydrophobic carbon resists moistening by the polar electrolytes, causing slow ion transmission and poor rate performance.^[^ ^74^ ^]^ Overall, carbon materials cannot effectively inhibit the shuttle effect.^[^ ^12^ ^]^ Excitingly, porous clays are a type of promising candidate for solving these issues. The hydrophilic Si—OH group of the clay motivates the electrolyte infiltration and enhances the rate performance. Additionally, the energy barrier for lithium ion diffusion of the clay/sulfur is lower than that of the carbon‐based electrodes.^[^ ^75^ ^]^

##### Clays for Li–S Battery Cathodes

**Figure 6:**
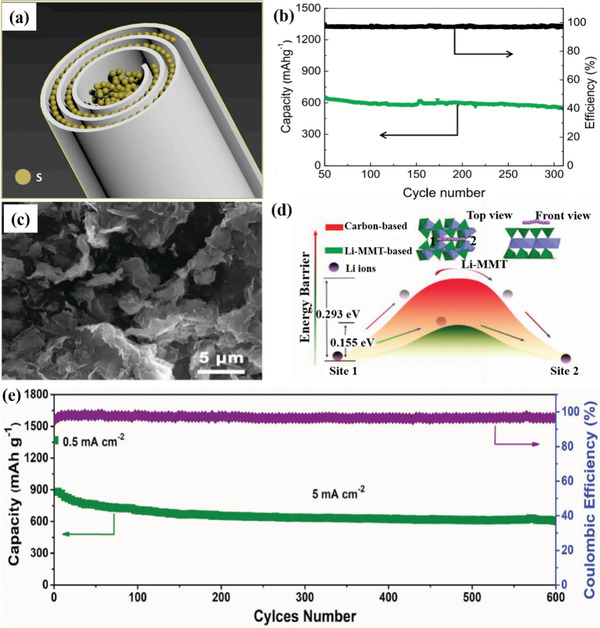
a) Sulfur nanoparticles confined inside halloysites. b) Cycling performances of sulfur/halloysites composite cathode.^[^ ^12^ ^]^ Reproduced with permission.^[^ ^12^ ^]^ Copyright 2020, Elsevier. c) SEM image of Li‐MMT. d) The energy barriers for lithium ion transportation of Li‐MMT and carbon‐based electrodes. e) Long‐term cycling performance of Li‐MMT/S cathode.^[^ ^75^ ^]^ Reproduced with permission.^[^ ^75^ ^]^ Copyright 2018,Wiley‐VCH.

In this section, recent advances of clays as cathodes for Li–S batteries is presented. Pei et al. prepared carbon skin wrapped halloysite/sulfur cathodes of Li–S batteries through solution impregnation.^[^ ^12^ ^]^ As shown in **Figure** **6**a, sulfur nanoparticles were captured in both inside and outside surface of the halloysite nanotubes (HNTs) with a loading amount up to 80 wt%. The layered architecture of the halloysite provided a large specific surface area (44.8 m^2^ g^−1^) to confine sulfur nanoparticles and buffer the volumetric expansion. The hollow architecture of halloysites offered adequate space to permit the volumetric change of entrapped sulfur nanoparticles and restrict their size up to the diameter of HNTs during lithiation/delithiation process. The increased tortuosity derived from the nanotube structure of natural halloysite inhibited the solvation of polysulfides and the shuttle effect. The HNTs contained abundant isolated domains to control the dissolution of polysulfides and increased the barrier of diffusion for the polysulfides to retard their migration. The outstanding composite structure contributed to the high capacity (657 mAh g^−1^ at 0.1C after 250 cycles) and stable cycling performance (capacity retention of 84% over 250 cycles), shown in Figure 6b.

Chen et al. decreased lithium ion diffusion barrier in a high content sulfur cathode through preparing Li‐MMT/S cathode for Li–S batteries.^[^ ^75^ ^]^ The surface area of the pure MMT was 62.23 m^2^ g^−1^. After incorporating with 80 wt% sulfur powders, the surface area decreased to 34.91 m^2^ g^−1^. The Li‐MMT/S cathodes exhibited excellent wettability, representing an almost indiscernible water contact angle once contacted with the electrolyte. As shown in Figure 6c, Li‐MMT has a typical nanosheet structure, permitting fast lithium ions transport and effective penetration of electrolytes into the cathodes. The fast lithium ion transport with high current density inhibited the growth of lithium dendrites. The diffusion mechanism of lithium ions in the interlayer of Li‐MMT has been investigated via density functional theory (DFT) calculation. As illustrated in Figure 6d, the energy barrier of Li‐MMT was 0.155 eV, is less than that of the carbon‐based electrodes, elucidating faster lithium ion diffusion around the Li‐MMT structure. The ultra‐wide interlayer distance and a low energy barrier of Li‐MMT/S cathodes promoted the lithium ion diffusion rate, resulting in stable cycling and excellent rate performance (capacity of 1000 mAh g^−1^ at 0.5 mA cm^−2^ and 717 mAh g^−1^ at 5 mA cm^−2^). Under a high current density test, Li‐MMT/S cathodes exhibited a high capacity of more than 700 mAh g^−1^ over 600 cycles at a large current density of 5 mA cm^−2^ as shown in Figure 6e.

**Figure 7:**
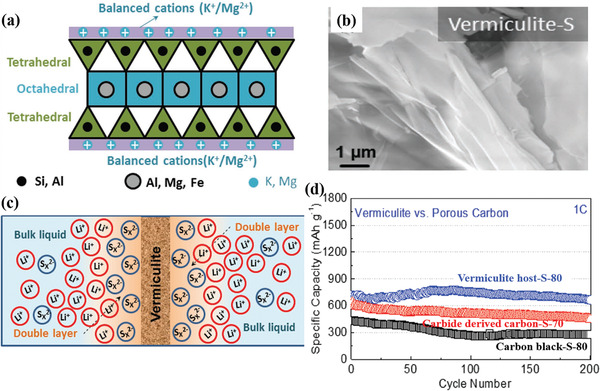
a) The crystalline structure of vermiculite. b) The SEM image of vermiculite–S. c) The double electric layer effected by vermiculite. d) Cycling performance over 200 cycles at 1C of vermiculite–S.^[^ ^76^ ^]^ Reproduced with permission.^[^ ^76^ ^]^ Copyright 2019, Wiley‐VCH.

Similarly, Wu et al. infiltrated sulfur into the porous vermiculite host and used it as the cathode for Li–S batteries.^[^ ^76^ ^]^ The crystalline structure of vermiculite is shown in **Figure** **7**a. The morphological characteristic of vermiculite–S is shown in Figure 7b. The expanded vermiculite is a commercial product that is relatively inexpensive (≈£80 per ton). The cations of vermiculite surface (such as Mg^2+^, Ca^2+^, K^+^, Li^+^, and Na^+^) could help absorb the anions of the polysulfides (S*_n_* ^2−^), thus preventing polysulfides from dissolution. The effect of absorption facilitated the formation of double electric layers, as exhibited in Figure 7c. The aggregation of lithium ions at the side of the electrolyte promoted charge transmission and ion diffusion. Hence, vermiculite‐S cathodes demonstrated an outstanding rate capability and cycling stability (the capacity retention of 93% at 1C within 200 cycles), as shown in Figure 7d.

Above all, clay/S cathodes for Li–S batteries have many advantages as follows: 1) The clay provides enough interspace to accommodate the volume expansion of sulfur cathode. 2) Clays with different morphology confine the sulfur cathode and suppress the dissolution and shuttle effect of polysulfides. 3) Clay/S cathodes permit free transportation of lithium ions, resulting in efficient and straightforward ion diffusion. Therefore, natural clays are appropriate hosts for sulfur to obtain remarkable electrochemical properties of Li–S batteries.

##### Clays for ZIB Cathodes

**Figure 8:**
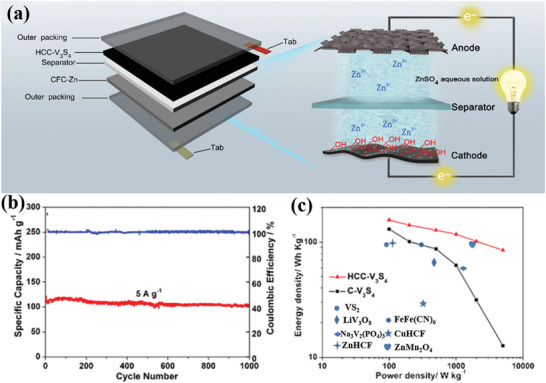
a) Schematic illustration of HCC‐V_3_S_4_//CFC−Zn device and component.^[^ ^77^ ^]^ b) Cycling performance and Coulombic efficiency. c) Energy density versus power density plots of the HCC‐V_3_S_4_//CFC‐Zn and C‐V_3_S_4_//CFC‐Zn devices prepared in this work in contrast with other reported work. Reproduced with permission.^[^ ^77^ ^]^ Copyright 2019, American Chemical Society.

Clay cathodes can be used not only in the field of Li–S batteries, but also as ZIB cathodes. By utilizing the wettability modification of the clay‐based materials, Liu et al. constructed a novel cathode for ZIBs, consisting of acid‐treated halloysite, carbon nanotubes and V_3_S_4_ materials (denoted as HCC‐V_3_S_4_).^[^ ^77^ ^]^ The aqueous ZIB was assembled using the HCC‐V_3_S_4_ as the cathode and the Zn nanosheet/carbon fiber cloth (CFC) as the anode, as shown in **Figure** **8**a. The adsorption energy of H_2_O for the SiO_2_ derived from halloysite was calculated utilizing DFT. The adsorption energy for the SiO_2_ was low (−0.69 eV), proving the remarkable hydrophilicity of SiO_2_. Natural halloysite promoted the hydrophilicity of Zn^2+^ electrolyte and improved the transmission rate of electrolyte ions. The carbon nanotubes increased the electronic conductivity and charge transfer kinetics. Therefore, the aqueous zinc ion battery displayed a high capacity retention (102 mA h·g^−1^ at a high current density of 5 A·g^−1^ over 1000 cycles) and a remarkable energy density of 155.7 W h·kg^−1^, along with high power density of 5000 W·kg^−1^, displayed in Figure 8b,c.

##### Clays for CIB Cathodes

CIBs are a type of promising energy storage device on account of their large theoretical volumetric energy density (up to 2500 Wh L^−1^) and substantial reserves of chloride‐containing materials.^[^ ^78^ ^]^ Specially, unlike LIBs, CIBs have the dendrite‐free feature, contributing to a safer and large‐scale energy storage device. However, the short cycling lifespan and unstable structure of cathode materials impede their industrialized application.^[^ ^79^ ^]^ Yin et al. prepared NiFe‐based layered double hydroxide (LDH) intercalated by chloride ions as cathode materials for CIBs.^[^ ^80^ ^]^ LDHs are a type of 2D brucite‐like anionic clays. LDHs with 2D diffusion pathways had high anion‐exchange capacity with reversible anion intercalation/de‐intercalation. The particular topochemical feature resulted in structural stability of LDHs. The chloride ions reversibly shuttled between electrodes, accompanied by redox reactions of Ni^2+^/Ni^3+^ and Fe^2+^/Fe^3+^ in the LDHs during the electrochemical cycling. The NiFe‐Cl LDH cathode materials exhibited a high specific capacity of 350.6 mAh g^−1^ and a long lifetime of over 800 cycles at a current density of 100 mA g^−1^.

To conclude this section of clay‐based cathodes, compared to traditional carbon cathodes of Li–S batteries, clay‐based cathodes effectively enhance the electron conductivity and inhibit the shuttle effect. As for aqueous ZIBs, clay‐based cathodes improve the hydrophilicity of Zn^2+^ electrolytes and boost the diffusion rate of electrolyte ions. In CIB systems, clay‐based cathodes improve the structural stability and cycling lifespan.

#### 4.1.3 Separators

Recently, some sudden explosions of mobile phones and electric vehicles have raised serious concerns.^[^ ^81^ ^]^ The explosive accidents occurred frequently because of the short circuit caused by the breakage of the traditional polyethylene (PE) and polypropylene (PP) separators used under extreme conditions, such as crash, puncture, and high temperature.^[^ ^82^ ^]^ As a result, the separators of batteries require the features of thermal stability, good mechanical strength and flame retardance, in case of an internal short circuit.^[^ ^83^ ^]^ Additionally, separators used in batteries should also possess porous structure, good wettability for electrolytes and excellent ionic conductivity and electron insulativity.^[^ ^84^ ^]^ Although polyolefin‐based separators have good mechanical toughness and low cost, it tends to shrink under unexpected high temperature, resulting in inevitable short circuits.^[^ ^85^ ^]^ Thereby, it is critical to improve the stability of separators for batteries.

##### Clays for LIB Separators

**Figure 9:**
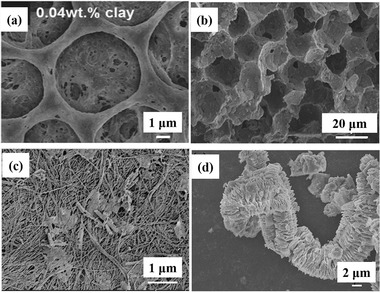
The SEM images of a) PVDF‐HFP/MMT clay.^[^ ^86^ ^]^ Reproduced with permission.^[^ ^86^ ^]^ Copyright 2015, Wiley‐VCH. b) MMT/P(VDF‐TrFE).^[^ ^87^ ^]^ Reproduced with permission.^[^ ^87^ ^]^ Copyright 2016, Elsevier. c) Bacterial cellulose/halloysite.^[^ ^88^ ^]^ Reproduced with permission.^[^ ^88^ ^]^ Copyright 2019, Springer. d) Porous expanded dickite clay.^[^ ^31^ ^]^ Reproduced with permission.^[^ ^31^ ^]^ Copyright 2019, IOP Publishing.

In this section, clay‐based separators for LIBs were systematically reviewed. Kim et al. directly dispersed 2D clay nanosheets (MMT) into poly(vinylidene fluoride‐co‐hexafluoropropylene) (PVDF‐HFP) through one‐step coating method to serve as composite separators for LIBs.^[^ ^86^ ^]^ The morphological characteristic of PVDF‐HFP/MMT is shown in **Figure** **9**a. The original PVDF‐HFP separators had large pores (several micrometers). After the incorporation of clay nanosheets, the amount of small pores with 50 nm–2 µm pore sizes in the composite separator increased. Thus, the multiscale pores of this composite separator could produce extra ionic diffusion pathways. Increasing the additive clays to 0.04 wt%, enabled the maximum ionic conductivity of composite separators to reach 1.49 × 10^−3^ S cm^−1^. Therefore, owing to the increase of the contact area between the clay nanosheets and the PVDF‐HFP separators, lithium ions could transport more easily. The increased ionic pathways and electrolyte uptake derived from the high porosity improved the electrochemical properties. The PVDF‐HFP/clay composite separators displayed enhanced discharge capacity retention above 85% after 100 cycles. Furthermore, the incorporation of 2D clay nanosheets enhanced the thermal stability of the separator.

Nunes‐Pereira et al. fabricated porous poly(vinylidene fluoride‐co‐trifluoroethylene) (P(VDF‐TrFE)) composite separators of LIBs modified with clays, zeolites, ceramics, and carbonaceous fillers.^[^ ^87^ ^]^ In contrast to pure polymer separators, the ionic conductivity, thermal stability, mechanical stability and comprehensive electrochemical performances of separators filled with fillers increased to a great extent. Impressively, the composite separators filled with 4% MMT clay had the best electrochemical performance among the fillers. The morphological characteristic of MMT/P(VDF‐TrFE) is shown in Figure 9b. The surface area, porosity and ionic conductivity of MMT/P(VDF‐TrFE) composite separators were 220–270 m^2^ g^−1^, 83%, and 3.6 × 10^−4^ S cm^−1^, respectively. The MMT with high surface area and porous structure positively improved the ionic diffusion and decreased the interfacial resistance. The half‐cells with the composite separators consisting of 4% MMT exhibited a specific capacity of 173 mAh g^−1^ at a current density of 0.1C.

Huang et al. prepared bacterial cellulose/HNT composite nanofiber separators through vacuum filtration.^[^ ^88^ ^]^ The morphological characteristic of bacterial cellulose/halloysite is shown in Figure 9c. The bacterial cellulose with electrolyte compatibility and thermostability is a promising candidate of separators for LIBs. The addition of HNTs further enhanced the ionic conductivity, electrolyte uptake and mechanical stability as well as reducing the interfacial resistance of the composite separators. The obtained composite bacterial cellulose/HNTs separator displayed a high tensile strength (84.4 MPa), an excellent porosity (83.0%) good electrolyte uptake (369%), favorable ionic conductivity (5.13 mS cm^−1^) and a broad potential window (2.5–4.0 V). The LIBs with the bacterial cellulose/halloysite separators exhibited a specific capacity of 162 mAh g^−1^ at a current density of 0.2C and cycling stability (95% of origin capacity after 100 cycles).

Liu et al. coated a porous expanded dickite clay on a cross‐linked nonwoven fabric as a separator for LIBs.^[^ ^31^ ^]^ The morphological characteristic of porous expanded dickite clay is shown in Figure 9d. The interactive sites between the Lewis acid of the expanded dickite clay and anions of the lithium salt electrolyte could release more lithium ions, which enhanced the ionic conductivity. Therefore, the expanded dickite coated composite separator demonstrated high electrolyte uptake (0.861 g cm^−3^) and ionic conductivity (3.157 mS cm^−1^). The porosity (61.6%) of the composite separators was greater than that of the commercial Celgard 2400 (40.8%). Employing this composite separator, LiFePO_4_/Li cells displayed an excellent discharge specific capacity (152 mAh g^−1^ at 0.5C) and high cycling stability (93.4% capacity retention after 200 cycles). In conclusion, the addition of clays enhanced the ionic conductivity, porosity, electrolyte uptake ability and mechanical stability of composite separators for LIBs. The application of clays for LIBs separators has great development potential. The pore‐forming agent endows clays as LIBs separators with adjustable pore size, improved the electrolyte uptake ability. In addition, mechanical and thermal stability should be measured quantitatively via systematic approaches.

##### Clays for Li–S Battery Separators

As for Li–S batteries, the separators should have the function of good lithium ion conductivity and an outstanding inhibiting effect of polysulfide migration.^[^ ^89^ ^]^ If the shuttle effect of polysulfides can be effectively suppressed, the electrochemical properties of Li–S batteries would be greatly enhanced.^[^ ^90^ ^]^ To settle the problems of the dendrite growth and the shuttle effects seen in Li–S battery, Xu et al. assembled 2D vermiculite sheets that served as the separator.^[^ ^91^ ^]^ The exfoliated vermiculite separators with a compact stacking structure was negatively charged, which inhibited the shuttle effect of polysulfides via electrostatic repulsion. The 2D vermiculite sheets with the interlayer structure permitted selective transportation of lithium ions, avoiding the shuttle effect in Li–S batteries. In addition, natural inorganic separators possessed strong Young's modulus (175 GPa), which restrained the penetration of lithium dendrites. The cell containing a vermiculite separator showed an excellent initial specific capacity of 1000 mAh g^−1^ and an average Coulombic efficiency of 90.3% after 50 cycles. Liu et al. employed porous dickite clays coated on the ethylene/propylene separators.^[^ ^92^ ^]^ The expanded dickite clays with porous structure were fabricated through the intercalation/deintercalation of urea into the interlayer of dickites with the existence of KClO_3_. The pore size of the expanded dickite was 0.3–1.2 µm. After modifying the organic separator of rechargeable hybrid aqueous batteries, the composite separators had a better porosity (67.1%), electrolyte uptake (1.378 g cm^−3^) and ionic conductivity (13.12 mS cm^−1^). The large pores and high porosity of composite separators with 90 wt% expanded dickite facilitated more pathways for ion transport, thus decreasing the concentration polarization during the charge/discharge process. Therefore, the composite separators with 90 wt% expanded dickite displayed remarkable electrochemical performances (132 mAh g^−1^ at 0.2C and 89 mAh g^−1^ at 4C).

In essence, the separator, as a vital component of the batteries, permits a fast diffusion of metal ions. Essentially, the separator is placed between the positive and negative electrodes, which can prevent batteries from internal short circuits. The composite separators modified by clays have attracted much attention, because 1) polyolefin separators amended by the clay efficiently avoid thermal shrinkage under the high temperature; 2) the good wettability of composite separators improves the electrolyte uptake due to its ameliorated wettability; 3) clays will enhance the ionic conductivity and reduce the intrinsic resistance of separators.

#### 4.1.4 Solid‐State Electrolytes

Alkali metal ion (Li^+^, Na^+^, K^+^) batteries are popular in the field of energy storage systems. As discussed above, traditional flammable organic electrolytes of alkali metal ion batteries have serious electrolyte leakage and safety problems.^[^ ^93^ ^]^ To tackle the above issues, solid‐state electrolytes have appeared as a type of promising alternative to organic electrolytes. Solid‐state electrolytes are classified as either inorganic or polymer electrolyte.^[^ ^94^ ^]^ Although inorganic solid electrolytes generally exhibit high ionic conductivity, the brittleness and the weak interfacial contact between the electrode and the electrolyte impede their further development.^[^ ^95^ ^]^ In comparison, solid polymer electrolytes are regarded as nonsolvent salt solutions in polymer materials.^[^ ^96^ ^]^ The solid polymer electrolytes possess mechanical stability and low electrode/electrolyte interfacial resistance (207 Ω cm^−2^).^[^ ^97^, ^98^ ^]^ Furthermore, solid polymer electrolytes can hinder the excessive growth of lithium dendrites and exhibit outstanding electrochemical stability. However, the ionic conductivity of solid polymer electrolytes is difficult to meet the demand for high power density.^[^ ^99^ ^]^ Overall, the organic‐inorganic composite solid‐state polymer electrolyte is a creative approach to improve the electrochemical properties.

**Figure 10:**
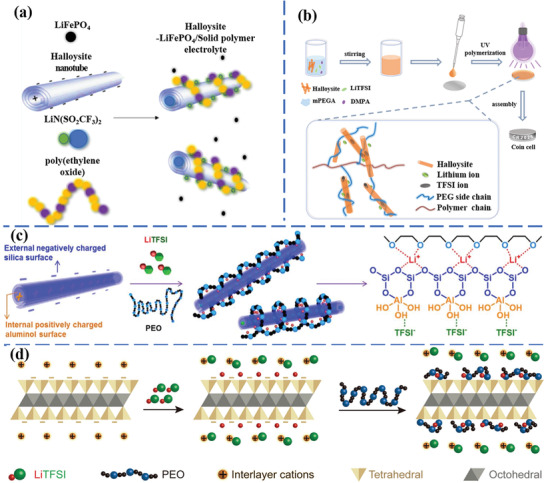
Schematic illustration for the preparation processes of a) halloysite‐LiFePO_4_ solid polymer electrolyte.^[^ ^100^ ^]^ Reproduced with permission.^[^ ^100^ ^]^ Copyright 2019, American Chemical Society. b) Halloysite clay composited methoxy poly(ethylene glycol) acrylate polymer electrolyte.^[^ ^14^ ^]^ Reproduced with permission.^[^ ^14^ ^]^ Copyright 2020, Elsevier. c) HNT modified solid polymer electrolyte.^[^ ^101^ ^]^ Reproduced with permission.^[^ ^101^ ^]^ Copyright 2017, Elsevier. d) Vermiculite sheets decorated solid polymer electrolyte.^[^ ^102^ ^]^ Reproduced with permission.^[^ ^102^ ^]^ Copyright 2018, Wiley‐VCH.

Zhu et al. fabricated effective halloysite/poly(ethylene oxide) (PEO)‐LiFePO_4_ solid polymer electrolyte for LIBs, as shown in **Figure** **10**a.^[^ ^100^ ^]^ The zeta potential of the halloysite could be explained by an outer layer of SiO_2_ with negative charge and the Al_2_O_3_ inner surface with positive charge. Thus, the TFSI^−^ anions of electrolytes would preferentially adsorb on the inner surface of the halloysite and the lithium ions would likely to adhere on the SiO_2_ surface. The lone‐pair electrons of the EO units acted with lithium ions on the surface of HNTs. The interactions between halloysites, lithium bis(trifluoromethanesulfonyl)imide (LiTFSI) and PEO caused the unique 3D architecture for lithium ion diffusion. The solid polymer electrolyte filler used in the halloysite improved the ionic conductivity (9.23 × 10^−5^ S cm^−1^ at 25 °C), lithium‐ion transference number (0.46) and electrochemical voltage window (5.14 V). The addition of HNTs and LiFePO_4_ contributed to the stable interfacial resistance, because they improved the compatibility as well as the charge transfer between the electrodes and electrolytes. The all‐solid‐state batteries contained the composite electrolyte had excellent cycling performances (capacity of 156 mA h g^−1^ after 100 cycles at 0.1C and 90.9% capacity retention).

Feng et al. prepared HNTs/methoxy poly(ethylene glycol) acrylate composite polymer electrolyte (HCPE) for lithium metal battery through UV triggered radical polymerization, as demonstrated in Figure 10b.^[^ ^14^ ^]^ The introduction of HNTs decreased the regularity and the ratio of crystalline regions of the polymers, which was beneficial for lithium ion diffusion. The unique structure of natural halloysite clay promoted the dissolution of lithium salt, resulting in an improvement in the ionic conductivity (5.62 × 10^−5^ S cm^−1^). The electrochemical stable voltage window of the solid‐state electrolyte was 5.28 V. The decomposition temperature of the solid‐state electrolyte was about 346 °C, indicating that the introduction of halloysite improved the thermal stability. Meanwhile, the obtained composite solid‐state electrolyte demonstrated its compatibility with Li metal and high electrochemical stability. The all‐solid‐state cell delivered a specific capacity of 138.3 mAh g^−1^ and a Coulombic efficiency of 98.1% at 0.2C.

Lin et al. added HNTs into solid polymer electrolytes to obtain all‐solid‐state Li–S batteries.^[^ ^101^ ^]^ In Figure 10c, the halloysite surface with opposite charges divided lithium salts into lithium cations and anions. The lithium ions were adhered on the outer clay surface with negative charges, and the anions were absorbed on the inner surface with positive charges. Owing to the addition of HNTs, the composite solid polymer electrolyte showed an exceptional ionic conductivity of 1.11 × 10^−4^ S cm^−1^, lithium ion transference number of 0.40 at 25 °C and could operate over the temperature range of 25–100 °C. The Li–S batteries with composite solid polymer electrolytes delivered a stable discharge capacity of 745 mAh g^−1^ after 100 cycles along with 87% capacity retention at 25 °C and 0.1C.

Figure 10d shows Tang et al. work of prepared 2D vermiculite nanosheets decorated PEO‐based solid polymer electrolyte.^[^ ^102^ ^]^ The 2D vermiculite could concurrently interact with the salt anions and the polymer segments to facilitate local amorphization and improve the lithium ion diffusion. The aligned vermiculite nanosheets in solid polymer electrolyte improved the ionic conductivity. The restacked vermiculite films were demonstrated to act as proton channels with high thermal stability up to 500 °C. This composite solid polymer electrolyte displayed high thermal stability, tensile strength (0.8 MPa), tensile modulus (13.1 MPa) and ionic conductivity (2.9 × 10^−5^ S cm^−1^ at 25 °C). Furthermore, the addition of clay vermiculite sheets into the solid polymer electrolyte decreased the flammability and enhanced the compatibility between the electrode and the electrolyte. The Li/LiFePO_4_ cells containing composite solid polymer electrolytes displayed the capacities of 159.9 mAh g^−1^ at 0.1C and 152.0 mAh g^−1^ at 0.5C.

Gel polymer electrolytes (GPEs) are another type of solid‐state electrolytes. GPEs solve the unsafe problems of organic liquid electrolytes, such as leakage, internal short circuit and flammability.^[^ ^81^ ^]^ Nonetheless, the disadvantages of GPEs such as limited low ionic conductivity, inferior mechanical properties, narrow electrochemical stability window and huge interfacial resistance between electrodes and GPEs inhibit their large‐scale development.^[^ ^103^ ^]^ To tackle the above issues, there are researches underway to enhance the comprehensive properties of GPEs. Hence, it is necessary to find new modification approaches of GPEs to realize excellent overall electrochemical performance. The cellulose acetate/poly‐L‐lactic acid/halloysite nanotube (CA/PLLA/HNT) composite GPEs used in LIBs were prepared by Zhu et al.^[^ ^104^ ^]^ The biodegradable PLLA polymer has mechanical stability and flexibility, rendering it potential skeleton materials for GPEs. However, the crystallization of PLLA caused poor electrochemical performance. The introduction of HNTs could suppress the crystallization behavior of PLLA and improve the ionic conductivity and the thermal stability of the GPEs. The composite GPEs demonstrated high porosity (83%), ionic conductivity (1.52 × 10^−3^ S cm^−1^), lithium‐ion transference number (0.45) and saturated electrolyte uptake ratio (600 wt%). The Li/GPE/LiCoO_2_ cells with CA/PLLA/HNT separators delivered a reversible discharge capacity of 125.2 mA h g^−1^ at 0.1C and a capacity retention of 93.1% after 50 cycles.

As a consequence, natural clays can decrease the crystallization regions and increase the lithium salt dissolution.^[^ ^105^ ^]^ The Si—O—Si groups of the clay surface attract lithium ions. The lithium ions can move freely outside of the clay layer surface and the ionic conductivity is improved. Moreover, natural clays work well in both solid‐state electrolytes and gel polymer electrolytes. Clays boost the comprehensive performance of electrolytes, including high thermal stability, satisfactory ionic conductivity, suitable lithium‐ion transference number, and great electrochemical stability. Furthermore, clay‐based solid‐state electrolytes have the potential to substitute the conventional electrolytes of other metal‐ion batteries, such as ZIBs, NIBs, and KIBs.^[^ ^106^ ^]^

In this section, we put forward an overview on the anodes, cathodes, separators and solid‐state electrolytes of clay‐based composites in rechargeable metal‐ion batteries. Through the different modified methods, the clay‐based materials obtained the anticipative performances. In the following sections, the performances of clay‐based composites for supercapacitors will be presented.

### 4.2 Clay‐Based Composites for Supercapacitors

Currently, supercapacitors are widely used for powering fast charge devices, due to their high power density (15 kW kg^−1^), short charge/discharge time (supercapacitors: 1–10 s vs LIBs: ≈ 600 s) and stable cycling performance (supercapacitors: > 30000 h vs batteries: > 500 h).^[^ ^107^, ^108^ ^]^ Similarly, the electrochemical performance of supercapacitors is mainly influenced by electrode materials, electrolytes and the working voltage.^[^ ^109^ ^]^ The vast majority of research has been oriented by the development of advanced structure of the electrode materials and the electrolytes with fast electron transmission and ion diffusion.

According the charge storage mechanism, a supercapacitor can be categorised into three types:^[^ ^110^ ^]^ 1) electrochemical double layer capacitors (EDLCs), 2) pseudocapacitors (PCs), and 3) hybrid capacitors (HCs). The mechanism of EDLCs is that the external charges of the electrode attract the opposite charged ions from the surrounding electrolytes, and these ions are attached to the electrode surface to form a double charged layer in the process of charging.^[^ ^111^ ^]^ EDLCs display a higher energy density (65–115 kJ kg^−1^ with the voltage window of 3–4 V) than other traditional capacitors due to the absorption of the ions from the electrolytes and the small charge separation distance.^[^ ^107^, ^112^ ^]^ EDLCs comprise an electrolyte, such as KOH, H_2_SO_4_, or Na_2_SO_4_, between the electrodes rather than the dielectric materials used in traditional capacitors. On the other hand, the charge‐storage mechanism of PCs is dominated by fast and reversible surface redox reactions (Faradaic processes).^[^ ^113^ ^]^ The capacitance of PCs is much higher than that of EDLCs because the pseudocapacitance can be generated from the surface redox reaction.^[^ ^114^, ^115^ ^]^ But the power property of PCs is inferior compared to that of EDLCs because the Faradaic process shows relatively sluggish kinetics during charge/discharge process, causing an inferior cycling stability and rate capability.^[^ ^116^ ^]^ As an emerging type of supercapacitors, HCs combine the advantages from both EDLCs and PCs.^[^ ^117^ ^]^

Various active electrode materials of supercapacitors are proposed, such as modified porous carbon, transition metal oxides/hydroxides, and conductive polymers.^[^ ^118^, ^119^, ^120^ ^]^ Nevertheless, for the electrode materials, they cannot concurrently meet the demands of the overall electrochemical performances, such as high ionic conductivity, high capacitance, reversible charge/discharge process and excellent energy and power density. Therefore, many endeavors have been made to solve the issues with developing multifunctional electrodes and electrolytes. The attractive features make the modified clays promising electrodes for the supercapacitors, owing to their high specific surface area, adjustability of positively and negatively charged active species, high ionic conductivity and thermal stability. However, the huge electrical resistance of clays impedes the application of clays as an electrode. Thus, the modification of conductive carbon‐based electrodes is a straightforward strategy. In addition, ionogels are nonflammable and nonvolatile, and provide high thermal and electrochemical stability.^[^ ^121^ ^]^ Utilizing ionogels as electrolytes is a persistent strategy to enable high‐voltage supercapacitors, which increases the energy density of supercapacitors. However, most ionogels are still too fragile to withstand severe temperature changes and large deformations, thus resulting in unstable operation.^[^ ^122^ ^]^ In order to solve the above problems, carbon nanotubes, graphene, and MXene sheets as fillers coupled with solid‐state polymers have served as ionogels. However, the compatibility between electrodes and ionogels is another vital factor.^[^ ^123^ ^]^ The clays also have been selected as the nanofillers of ionogels because of their easy dispersion property in ionic liquids and high ionic conductivity.

**Figure 11:**
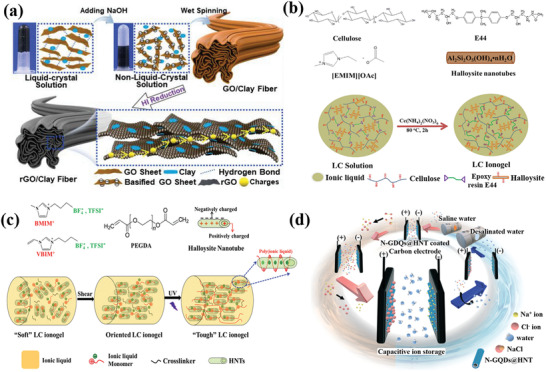
Schematic illustration for the preparation processes of a) rGO/clay electrode.^[^ ^124^ ^]^ Reproduced with permission.^[^ ^124^ ^]^ Copyright 2020, Elsevier. b) Cellulose/ionic liquid/halloysite ionogel.^[^ ^125^ ^]^ Reproduced with permission.^[^ ^125^ ^]^ Copyright 2018, Elsevier. c) Poly(ionic liquid)/halloysite ionogel.^[^ ^126^ ^]^ Reproduced with permission.^[^ ^126^ ^]^ Copyright 2019, Royal Society of Chemistry. d) N‐GQDs@HNT coated carbon electrode and the application in capacitive deionization.^[^ ^127^ ^]^ Reproduced with permission.^[^ ^127^ ^]^ Copyright 2020, Royal Society of Chemistry.

Chen et al. intercalated the clay nanofillers (laponite) into a graphene oxide electrode through a nonliquid‐crystal spinning method to improve the specific capacitance.^[^ ^124^ ^]^ The reduced graphene oxide/clay (rGO/clay) fibers were prepared via chemical reduction reaction, as shown in **Figure** **11**a. The graphene electrodes could not realise the theoretical capacitance due to the hydrophobicity feature and *π*–*π* stacking of graphene sheets. However, the addition of clays improved the porosity, ionic conductivity and affinity with the electrolytes for graphene electrodes. The obtained rGO electrodes with 20% clay loading possessed good mechanical strength (102.7 MPa), hydrophilicity (contact angle of 58.3°), ionic conductivity (11.5 S cm^−1^) and outstanding capacitive performance. The rGO/clay electrode exhibited a specific capacitance of 197.0 F g^−1^ at a current density of 0.2 A g^−1^. The flexible all‐solid‐state supercapacitor showed a superior energy density of 6.14 mWh cm^−3^ (5.24 mWh g^−1^) at a power density of 28.33 mWh cm^−3^ (24.17 mWh g^−1^).

Moreover, Guo et al. synthesized cellulose/ionic liquid/halloysite ionogels through a chemical crosslinking process as shown in Figure 11b, and the halloysite clay was regarded as the ionic conductive agent.^[^ ^125^ ^]^ The anisotropic halloysite clay provided multiple ionic transportation channels. The ionic conductivity of which has increased to the order of 1 mS cm^−1^. The addition of the halloysite significantly enhanced the mechanical stability of supercapacitors. The reason is that the surfaces of halloysites have many hydroxyl groups, which have robust interactions with the ‐OH groups of cellulose through hydrogen bonding. Due to the synergistic interactions between the ionic liquid, cellulose chains and halloysites, the thermal stability of the composite ionogels has also been enhanced. The specific capacitance of composite ionogels with 15% halloysites was 255.6 F g^−1^ at 120 °C, and the capacity loss was 7.5% after 5000 cycles.

Furthermore, Song's group designed anisotropic and ionic conductive poly(ionic liquid)/halloysite ionogels through shear‐induced orientation technology and UV photopolymerization method (Figure 11c).^[^ ^126^ ^]^ The HNTs could be easily dispersed in the polymer and organic solvents and aligned with opposite charges. The composite ionogels displayed a high modulus (≈26.7 MPa) and strong mechanical strength (≈4.4 MPa). Notably, the obtained ionogels showed excellent mechanical stability at the temperatures up to 200 °C. Similarly, proved by another reported work,^[^ ^125^ ^]^ due to the oriented charged HNTs, the flexible solid‐state supercapacitor displayed superior ionic conductivity (≈6 mS cm^−1^), high specific capacitance (277.4 F g^−1^ at 120 °C), and remarkable cycling performance (95.2% capacitance retention at 1 A g^−1^ for 5000 cycles).

Ganganboina et al. synthesised nitrogen doped graphene quantum dots@halloysite nanotubes (N‐GQDs@HNTs) electrode materials for capacitive deionization application,^[^ ^127^ ^]^ as shown in Figure 11d. The 1D structure of HNT offered a large specific surface area (45.4 m^2^ g^−1^) to load N‐GQDs as well as to absorb ions. In addition, the mesoporous structure of the HNTs shortened the diffusion distances to boost the electron and ion kinetics during charge/discharge processes. The incorporation of N‐GQDs into HNTs improved the electrical conductivity and decreased the interfacial contact resistance. The N‐GQDs@HNTs possessed numerous charge storage sites and high ionic conductivity, leading to the promotion of the charge transfer and ion diffusion. Therefore, the GQDs@HNTs electrodes demonstrated an excellent electric double layer characteristic with a remarkable specific capacitance of 335 F g^−1^ at 0.5 A g^−1^.

In summary, the addition of clays into supercapacitors improve the ionic conductivity and the affinity with electrolytes. More details of the clays used in energy conversion systems will be discussed in the following sections.

### 4.3 Clay‐Based Composites for Energy Conversion Systems

#### 4.3.1 Clay‐Based Materials for Solar cells

Solar cells provide clean and sustainable energy via conversion the solar power into the electric energy.^[^ ^128^ ^]^ Through decades of development, solar cells have experienced three stages:^[^ ^129^ ^]^ 1) the first generation of crystalline silicon solar cells, 2) the second generation of thin film solar cells, 3) the third generation of emerging photovoltaics. The third generation is a great improvement and turns into one of the most popular devices in the solar cells. Organic solar cells (OSCs), perovskite solar cells (PSCs) and dye sensitized solar cells (DSSCs) are typical candidates for the third generation.^[^ ^130^, ^131^, ^132^ ^]^

Nowadays, DSSCs, as one of the most promising solar cells, exhibit a high photoelectric conversion efficiency of ≈15%. The typical DSSCs consist of a working anode (TiO_2_), a counter electrode (Pt), a chemical dye and an electrolyte (I^−^/I_3_^−^).^[^ ^133^ ^]^ The fundamental working principle of DSSCs is as follows:^[^ ^134^ ^]^ 1) after the absorption of sunlight, the electrons of chemical dye change from the ground state into the excited state, 2) the electrons of dye in the excited state are injected into the conduction band of a semiconductor, 3) the electrolyte reduces the dye from oxidized states to fulfill the regeneration of the dye, 4) the electrons from the conduction band pass through the semiconductor to the external circuit, generating electrical current in the solar cells. Based on the above mechanism, photovoltaic materials of DSSCs with low production cost and high energy conversion efficiency need to be designed.^[^ ^135^ ^]^ Although DSSCs with liquid electrolytes have a 11–13% photoelectric conversion efficiency,^[^ ^136^ ^]^ liquid electrolytes show the problems of solvent leakage and the dye degradation limiting the further development of DSSCs. To substitute traditional liquid electrolytes, many of the solid‐state electrolytes have been developed and applied in DSSCs. However, the solar to electric power conversion efficiency of solid‐state electrolytes still needs to be improved. Using inorganic nanofillers as additives of solid‐state electrolytes could increase the conductivity, rheological properties and the interfacial contact of electrode/electrolyte. Natural clays served as nanofillers in the electrolytes can increase the ionic conductivity and diffusivity, thermal stability, and decrease the charge transfer resistance.^[^ ^137^ ^]^

Prabakaran et al. incorporated the delaminated MMT nanoplatelets into the host matrix PEO/poly(vinylidene fluoride‐co‐hexaflouropropylene) (PVDF‐HFP) that served as the hybrid polymer electrolyte membrane for DSSCs.^[^ ^138^ ^]^ The PEO/PVDF–HFP composites provided photochemical stability and mechanical strength. The 2D layered MMT nanoplatelets were exfoliated using aminopropyltrimethoxy silane (APS). The Si–O–Si, NH_2_ and CH_2_ groups of APS were loaded on the surface of MMT nanoplates, which may help to increase the amount of the intercalated polymer chains into the layered structure of nanoclays. Thus, there were more free volume of polymer electrolyte membranes to promote the ion diffusion. The incorporated layered MMT enhanced the ionic conductivity (up to 2.52 × 10^−3^ S cm^−1^), improved the energy conversion efficiency to 3.8%, and decreased the interfacial charge transfer resistance to 20 Ω. The photovoltaic performance of the composite polymer electrolyte membrane displayed an improved open circuit voltage of 0.73 V and a short circuit current of 7.7 mA cm^−2^ under an illumination of 100 mW cm^−2^. Wang et al. added the nitrate‐hydrotalcite nanoclay into the electrolyte to buffer the protonation process between the photoanode and the electrolyte.^[^ ^139^ ^]^ Due to the swelling ability, the ion exchange capacity and rheological properties, clays played a significant role to form gel electrolytes for quasi‐solid‐state DSSCs. The nitrate‐hydrotalcite nanoclay acts as an alkaline buffering source, resulting in the increase of conduction band energy. The equation of Fermi energy can be expressed as Equation (10)

(10) $$E_{F}=E_{C}\;+kT\ln\left(\frac{n_{c}}{N_{c}}\right)$$

where *E*_F_ is the Fermi energy, *E*_C_ is the conduction band energy, *kT* is the thermal energy, *n*_c_ is the electron concentration, and *N*_C_ is the effective concentration of accessible electronic states in the conduction band. The conduction band shifted to a superior energy level, causing an increase of Fermi level. The increase of open‐circuit potential (846 mV, nanoclay gel electrolyte in acetonitrile) was because of the change in Fermi energy. The additive nitrate‐hydrotalcite in the electrolyte not only protected the electrolytes from leaking, but also promoted the conversion efficiency to 9.6% under one‐sun illumination. Furthermore, Pedro et al. employed the artificial hydrotalcite clay as nanofillers into the electrolyte.^[^ ^140^ ^]^ The advantages of the hydrotalcite clay were not only solidifying the liquid electrolytes resulting in more stable quasi‐solid‐state electrolytes, but also improving the light scattering and ionic conductivity. The quasi‐solid electrolyte with 9 wt% nanoclay exhibited a high open‐circuit voltage (813 mV). The short circuit current density dropped to 18.9 mA cm^−2^ due to the existence of the hydrotalcite clay, which was attributed to the upward shift in the conduction band that decreased the electron injection. The clay addition agent in the electrolytes rendered stable solar cells under a sunlight test for over 1000 h and obtained a high photoelectric conversion efficiency of 10.9%. Costenaro et al. investigated the effect of saponite particles sizes on DSSC performances.^[^ ^141^ ^]^ The saponite clays with different particle size and spatial lamellae structure were used as additives for quasi‐solid electrolytes in DSSC. The saponite with the smallest particle size (50 nm) had an energy conversion efficiency of 8%.

To briefly conclude, the addition of clays into solid‐state electrolytes of solar cells enhanced the photoelectric conversion efficiency. In the following section, clay‐based materials used in the fuel cells will be introduced in detail.

#### 4.3.2 Clay‐Based Materials for Fuel Cells

Fuel cells convert chemical energy into electrical energy through redox reactions.^[^ ^142^ ^]^ In contrast to traditional combustion devices, fuel cells have no noise pollution and emit negligible harmful gases. Environmentally friendly fuel cells are classified into several types: hydrogen fuel cells (HFCs), direct methanol fuel cells (DMFCs), alkaline fuel cells (AFCs), polymer electrolyte membrane fuel cells (PEMFCs), solid oxide fuel cells (SOFC), microbial fuel cells (MFCs) and phosphoric acid fuel cells (PAFCs).^[^ ^143^ ^]^ Fuel cells are composed of anodes, cathodes, electrolyte membranes and current collectors. The electrolyte membrane is an essential component, which isolates the anode and cathode chambers and supports the ionic transportation between these two chambers.

Recently, electrolyte membranes have been extensively exploited, including ion exchange membranes (IEM), porous size‐selective membranes, and nanocomposite membranes.^[^ ^144^ ^]^ These membranes have some restrictions for practical application such as poor ionic transportation, unstable mechanical properties under high temperature, pH difference (4.4–10.3) between the anode and the cathode chambers and expensive cost (£65–300 m^−2^).^[^ ^145^, ^146^ ^]^ Many researchers have incorporated inorganic clay nanomaterials to solve these problems. Ishiyama et al. fabricated an anionic clay mineral (Mg‐Al layered double hydroxide intercalated with CO_3_^2−^) served as an electrolyte for fuel cells.^[^ ^147^ ^]^ The ionic conductivity of anionic clay mineral electrolyte was 2.2 × 10^−5^ S cm^−1^ and 1.4 × 10^−3^ S cm^−1^ at 20 and 80 °C, respectively. The anionic clay enhanced the working temperature to 80 °C and delivered high thermal stability. The anionic clay mineral showed high stability in alkaline solution (5M NH_4_OH + 1M KOH) and high temperature (80 °C) as an electrolyte separator.

**Figure 12:**
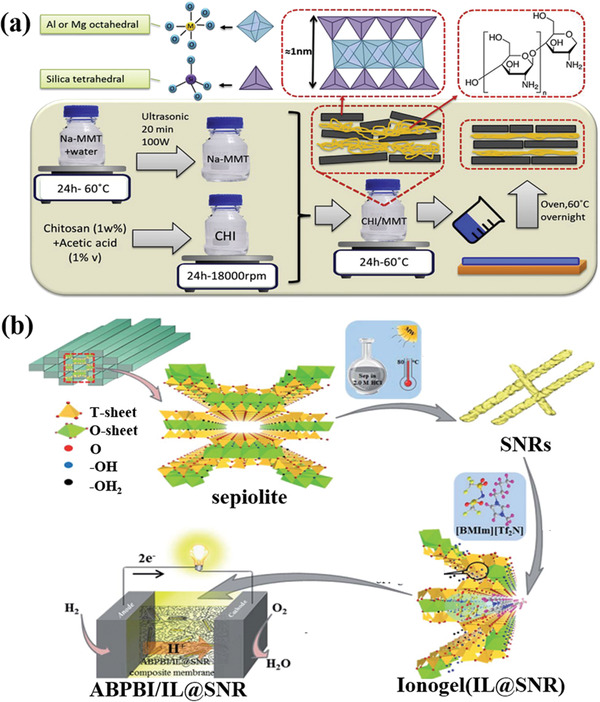
Schematic illustration for the preparation processes of a) CHI/MMT.^[^ ^148^ ^]^ Reproduced with permission.^[^ ^148^ ^]^ Copyright 2020, Elsevier. b) ABPBI/IL@SNRs.^[^ ^149^ ^]^ Reproduced with permission.^[^ ^149^ ^]^ Copyright 2019, Royal Society of Chemistry.

In **Figure** **12**a, the chitosan (CHI)/MMT nanocomposites were assembled as the electrolyte membrane of the MFCs by Yousefi et al.^[^ ^148^ ^]^ CHI is a nontoxic biopolymer with biocompatibility and film‐forming ability. However, the gas barrier, mechanical and thermal stability of CHI are not adequate for electrolyte membranes. The addition of MMT could improve the ionic conductivity and mechanical reinforcement of the composite electrolyte membranes. The oxygen transfer coefficient of CHI/MMT was decreased to 9.1 × 10^−6^ cm S^−1^. The ceramic membrane improved the electric double layer capacitance and decreased the charge transfer resistance to 28.88 Ω between electrodes, due to the promoted proton conductivity up to 222.73 µS cm^−1^ of the clay modified membrane. The Coulombic efficiency, power and current densities of the CHI/MMT were 86.97 ± 13.2%, 229.12 ± 18.5 mW m^−2^ and 1422.22 ± 41.2 mA m^−2^, respectively.

Zhang et al. prepared poly(2,5‐benzimidazole)/imidazolium ionic liquid@silicon nanorods (ABPBI/IL@SNRs) through in situ methods,^[^ ^149^ ^]^ as shown in Figure 12b. The SNRs derived from the acid‐treated natural sepiolite clay where the magnesium of sepiolite was removed by the hydrothermal treatment and the microwave irradiation. The 1D SNRs had a large specific surface area (252.88 m^2^ g^−1^) and a hierarchical porous structure. The embedded IL@SNR ionogels in the ABPBI substrate were regarded as proton conductors to offer extra proton transfer routes. The composite ABPBI/IL@SNRs membranes in fuel cells significantly enhanced the proton conductivity (0.01 S cm^−1^) over a broad temperature range (40–180 °C). The proton exchange membrane fuel cells delivered a high power density of 0.15 and 0.28 W cm^−2^ at 80 and 180 °C, respectively.

As discussed, clay‐based composites for energy conversion systems improved the conversion efficiency of solar cells and fuel cells. Particularly, natural clays served as nanofillers in the polymer electrolytes increased ionic conductivity and thermal stability.

## 5 Conclusions and Outlook

In this review, we have summarized cutting‐edge work and recent progress of the clay‐based materials for energy storage and conversion applications. First, the distribution, price, composition, physical and chemical properties, and conventional applications of natural clays were introduced in detail. Then, the structure, classification and modification methods of clays were discussed. Finally, an overview and the recent progress of clays and clay derivatives served as electrodes, electrolytes, separators, nanofillers in the fields of LIBs, Li–S batteries, ZIBs, CIBs, supercapacitors, solar cells, and fuel cells were provided.

Natural clay materials have many advantages in the field of energy storage and conversion, attributing to the following pivotal points: 1) Low cost and abundant reserves are the most competitive advantages of the clays; 2) Clay minerals have porous structures and high specific surface area. Significantly, after the modification process, tunable pore structures are obtained; 3) Clays have high thermal stability, mechanical stability and fire resistance. Theoretically, they can be used for large‐scale energy devices. 4) Owing to the Si—OH groups of the clay surface, clays tend to be hydrophilic in nature, which enhance the aqueous electrolyte uptake for specific energy applications. 5) Due to electrostatic interaction, some cations and polar water molecules are absorbed between the interlayers of the clay. Cations combining with water molecules form hydrated ions that can move between the interlayers. Therefore, clays have a good ionic conductivity, which improves the diffusion kinetics for energy devices.

However, clay minerals are brittle, 1D nanorods and 2D nanosheets of different forms of the clay are prone to aggregate. Therefore, further actions should be taken to improve the toughness and dispersibility of clay nanostructures, such as introducing long polymer chains into the clay matrix. Specially, clay‐based materials, due to their good ionic conductivity and insulative nature for electrons, are often sufficient as the separators for batteries, solar cells and fuel cells. However, as an electrode material, it needs not only good ionic conductivity but also excellent electronic conductivity. Thus, clay‐based electrodes are always decorated with carbon or conductive polymers. Particularly, clay‐based anodes are prone to expand or shrink during the charge/discharge process as the anodes for LIBs. It is a good approach to find a binder with great stretchability or form protective core‐shell structures to inhibit the severe volumetric change of clay‐based anodes.

**Figure 13:**
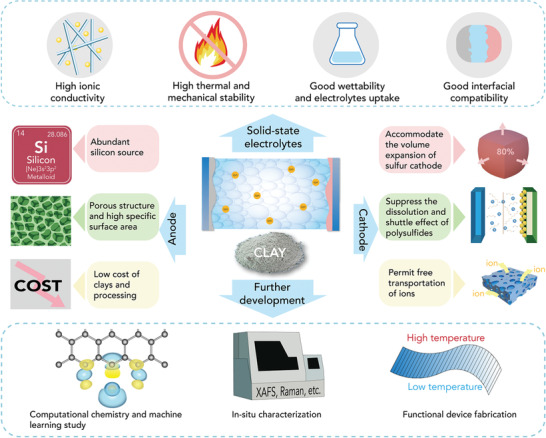
The advantages and further developments of clay‐based materials in the application of electrodes, electrolytes, and separators.

The research of clay‐based materials has promoted the development of electrodes, electrolytes and separators with novel morphology and superior performance, as shown in **Figure** **13**. The magnesium, iron, calcium, sodium, and potassium oxides occupy minor proportions of different types of clays, they may have unavoidable impacts on the performance when the natural clays are used as the electrode materials, for example as LIB anodes. The impact of these metal oxides should be investigated thoroughly in the future research. Notably, the specific reaction mechanisms, stability of structures and the performance evaluation of the clays should be further studied under both room temperature and the high/low temperature.

As for the modification methods of clay‐based materials, the widely used method is that natural clays are mostly leached by acid to remove impurities. However, the acid leaching only removes the general impurities, such as metal oxides and silts, moreover, different acid leaching and their influences on the final products are less investigated. The purified materials and different types of the acid should be evaluated systematically in future work. Moreover, when the clays served as separators or electrolytes, the long polymer chains introduced into the clay matrix extend the interlayer distance and improve the overall performance, but their compatibility with different types of clays are less considered. The types of polymers and the proportions of polymers and clays should be demonstrated in the future research. Besides, clay‐based composites for energy conversion systems improved the conversion efficiency of solar cells and fuel cells. Particularly, the addition of clay nanofillers into solid‐state polymer electrolytes enhanced the ionic conductivity, thermal and mechanical stability. More importantly, clay‐based electrodes modified by the flexible substrates, such as carbon fiber cloth or conductive long polymer chains, will improve their toughness, which can be used for the application in portable and wearable electronics. When the clays served as anodes, the magnesiothermic reduction or aluminothermic reduction are adopted to extract interconnected Si nanoparticles from the clays, which are relatively risky and complex involving high temperature and complicated facilities. The optimized and relatively mild methods to obtain clays can be fulfilled by physical methods, such as ball milling. Thus, developing facile preparation methods for clay‐based materials can improve the production efficiency and decrease the cost.

For different applications, we need to adopt suitable modification methods to realize the greatest advantages from clays. Notably, the detailed mechanisms of how clays serve as electrodes, electrolyte fillers, separators are less explored. The structures of natural clays are relatively complicated, therefore, it is hard to optimize the electrochemical performances and the fine adjustment and the reasonable selection of specific clays are needed for further modification. For examples, silicon anodes with different dimensions could be obtained by different types of natural clays. Therefore, the study by both in‐situ and ex‐situ spectroscopic and microscopic characterization of clays‐based energy materials should be carried out. The precise characterization on the structural analysis, electrochemical properties and mechanisms are necessary for identifying the active structures of clay‐based materials. The advanced technologies such as cryo–electron microscopy (cryo‐EM), X‐ray absorption fine structure (XAFS) analysis and in‐situ transmission electron microscope (TEM) are suggested to provide more details on the structure of clay‐based materials. Computational chemistry work will help to elucidate beneficial structures of clay‐based energy materials. For example, DFT calculations can provide information on electronic structures, defect formation energy, stable voltage range, theoretical capacity, ion transport kinetics, phase transition during ion (de)intercalation process of clay‐based materials for energy storage application. DFT can be used to study the thermodynamic free energy changes of chemical reaction of clay‐based materials for fuel cells and the polarizabilities, optimization geometry, conformational analysis, and photovoltaic analysis of clay‐based materials for solar cells. DFT calculations are crucial for mechanistic understanding and deepening the current understanding for energy storage and conversion fields. In addition, the COMSOL Multiphysics software can be used to simulate the distribution of electric field and ions in space and the surface current density of the clay‐based electrode materials. With persistent global efforts, it is anticipated that there will be more breakthroughs in clay‐based energy materials in the future. Indeed, we are confident that the inexpensive clay‐based energy materials will play an extremely essential role in cutting‐edge energy technologies.

## Conflict of Interest

The authors declare no conflict of interest.
